# Practical spectrophotometric assay for the *dapE*-encoded *N*-succinyl-L,L-diaminopimelic acid desuccinylase, a potential antibiotic target

**DOI:** 10.1371/journal.pone.0196010

**Published:** 2018-04-26

**Authors:** Tahirah K. Heath, Marlon R. Lutz, Cory T. Reidl, Estefany R. Guzman, Claire A. Herbert, Boguslaw P. Nocek, Richard C. Holz, Kenneth W. Olsen, Miguel A. Ballicora, Daniel P. Becker

**Affiliations:** 1 Department of Chemistry and Biochemistry, Loyola University Chicago, Chicago, Illinois, United States of America; 2 Regis Technologies, Inc., Morton Grove, Illinois, United States of America; 3 The Center for Structural Genomics of Infectious Diseases, Computation Institute, University of Chicago, Chicago, Illinois, United States of America; 4 Department of Chemistry, Marquette University, Milwaukee, Wisconsin, United States of America; University of East Anglia, UNITED KINGDOM

## Abstract

A new enzymatic assay for the bacterial enzyme succinyl-diaminopimelate desuccinylase (DapE, E.C. 3.5.1.18) is described. This assay employs *N*^6^-methyl-*N*^2^-succinyl-L,L-diaminopimelic acid (*N*^6^-methyl-L,L-SDAP) as the substrate with ninhydrin used to detect cleavage of the amide bond of the modified substrate, wherein *N*^6^-methylation enables selective detection of the primary amine enzymatic product. Molecular modeling supported preparation of the mono-*N*^6^-methylated-L,L-SDAP as an alternate substrate for the assay, given binding in the active site of DapE predicted to be comparable to the endogenous substrate. The alternate substrate for the assay, *N*^6^-methyl-L,L-SDAP, was synthesized from the *tert*-butyl ester of Boc-L-glutamic acid employing a Horner-Wadsworth-Emmons olefination followed by an enantioselective reduction employing Rh(I)(COD)(*S*,*S*)-Et-DuPHOS as the chiral catalyst. Validation of the new ninhydrin assay was demonstrated with known inhibitors of DapE from *Haemophilus influenza* (*Hi*DapE) including captopril (IC_50_ = 3.4 [± 0.2] μM, 3-mercaptobenzoic acid (IC_50_ = 21.8 [±2.2] μM, phenylboronic acid (IC_50_ = 316 [± 23.6] μM, and 2-thiopheneboronic acid (IC_50_ = 111 [± 16] μM. Based on these data, this assay is simple and robust, and should be amenable to high-throughput screening, which is an important step forward as it opens the door to medicinal chemistry efforts toward the discovery of DapE inhibitors that can function as a new class of antibiotics.

## Introduction

**Fig 1:**
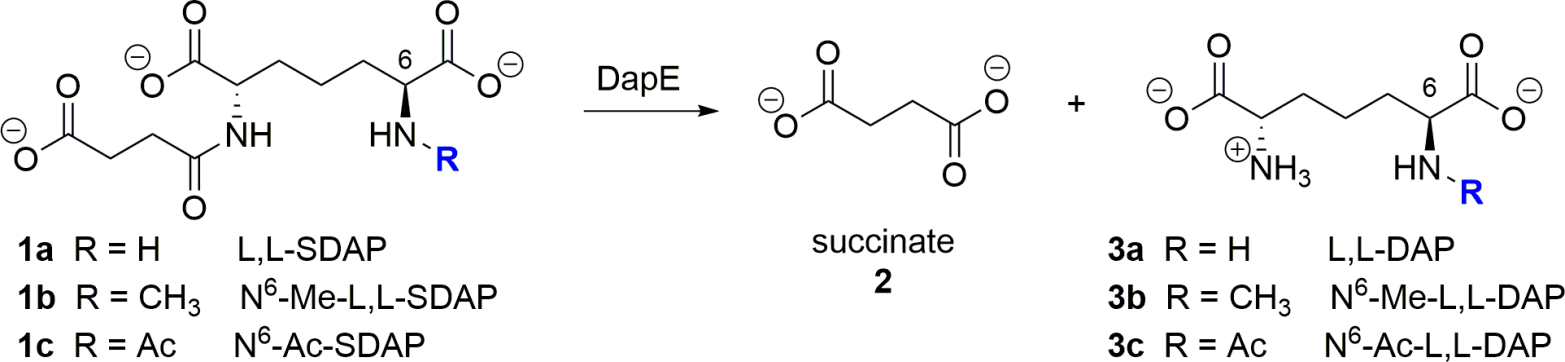
Hydrolysis of L,L-SDAP and analogs by *Hi*DapE. L,L-SDAP (**1a**) and analogs *N*^6^-methyl SDAP (**1b**) and *N*^6^-acetyl-SDAP (**1c**) with formation of hydrolysis products succinate (**2**) and L,L-diaminopimelic acid derivatives (**3a-c**). Enzyme-mediated hydrolysis was not observed for *N-*acetyl analog **1c** which would afford **3c**.

The sharp increase in mortality and morbidity due to rising bacterial infections caused by antibiotic-resistant bacteria[1] underlines the need to discover previously-unexplored enzymes as novel antibiotic targets with the concurrent goal of discovering and developing new molecular drug scaffolds. For example, invasive methicillin-resistant *Staphylococcus aureus* (MRSA) is a serious and growing health problem.[2] Several newly discovered strains of MRSA show antibiotic resistance even to vancomycin, which has been considered for decades as the standard for the treatment of systemic infections.[3] An attractive bacterial target that is present in all Gram-negative and most Gram-positive bacteria is the *dapE*-encoded *N-*succinyl-L,L-diaminopimelic acid desuccinylase (DapE, E.C. 3.5.1.18),[4] which is a member of the lysine biosynthetic pathway in bacteria that provides lysine and *meso*-diaminopimelate (m-DAP),[5] both of which are important for peptidoglycan cell-wall synthesis. DapEs catalyze the hydrolysis of *N-*succinyl-L,L-diaminopimelic acid (L,L-SDAP) to succinate and L,L-diaminopimelic acid (L,L-DAP) (Fig 1). Deletion of the DapE gene is lethal to *Helicobacter pylori* and *Mycobacterium smegmatis*, demonstrating the indispensable role of this enzyme in bacterial survival, and ultimately in pathogenesis in the human host.[6,7] Furthermore, lack of a similar pathway in humans suggests that selective inhibition of DapE may be toxic to bacteria but not to human hosts, making it a promising target for antibiotics with a new mechanism of action.[4]

Previously, it was shown that DapEs exhibit >60% of their maximal activity towards L,L-SDAP when only one Zn(II) ion is present in its active site,[8] with the tighter binding site confirmed by high-resolution X-ray crystallography. These structures have enabled further refinement of a mechanistic hypothesis of amide bond cleavage by DapE enzymes, which have in turn facilitated inhibitor identification.[9] A small, focused screen of compounds containing zinc-binding groups identified the thiol-containing ACE inhibitor captopril as a low micromolar competitive inhibitor (IC_50_ = 3.3 μM) of the DapE from *Haemophilus influenza* (*Hi*DapE) along with several other small molecule inhibitors including 3-mercaptobenzoic acid (IC_50_ = 35 μM), phenylboronic acid (IC_50_ = 107 μM), and 2-thiopheneboronic acid (IC_50_ = 92 μM).[10] A high-resolution (1.8 Å) X-ray crystal structure of captopril bound to the DapE from *Neisseria meningitidis* (*Nm*DapE) revealed a thiolate-bridged dinuclear Zn(II) active site[11] and provided a model for *in silico* approaches to identifying potential inhibitors of DapEs.[12]

The most widely used DapE assay monitors amide bond cleavage of L,L-SDAP spectrophotometrically at 225 nm.[10] While this assay is simple and reliable for simple inhibitors, it cannot be used to test potential inhibitors that absorb strongly in the UV region, precluding its use in testing many of the preferred medicinal chemistry leads and analogs. While several other assays have been developed for evaluating inhibitors of DapEs, all suffer from being technically troublesome and/or difficult to reproduce.[13] For example, Gelb *et al*.[14] took advantage of the fact that L,L-DAP reacts somewhat faster with ninhydrin than L,L-SDAP; however, this assay suffers from poor reproducibility. Two alternative assays were also reported, the first of which employed ^14^C-labeled L,L-SDAP while the second was a coupled assay that utilizes porcine succinate thiokinase and inositol triphosphate to convert liberated succinate to succinyl-CoA (CoA = coenzyme A) and inositol diphosphate. The inositol diphosphate was then detected by its reaction with phosphoenolpyruvate to yield liberated pyruvate, itself being detected spectrophotometrically using lactate dehydrogenase. The ^14^C-labeled substrate assay requires working with radioactivity, necessitating extra safety protocols and waste disposal issues, while the coupled assay is cumbersome and expensive while also being technically difficult and therefore difficult to reproduce, although succinate can now be determined by commercially-available kits that use proprietary coupled enzyme reactions, either following color development at 450 nm or detecting bioluminescence. However, the presence of secondary enzymes gives rise to the possibility of false positives through inhibition of the coupled enzyme, and the kits are not cost-effective for higher-throughput assays. Moreover, none of these assays are amenable to high-throughput screening studies, a necessity for the discovery of new lead compounds with antibacterial properties.

Recognizing the ease and reliability of ninhydrin-based assays to detect primary amino groups, such as that formed upon the hydrolysis of L,L-SDAP and the importance of the *N*^*6*^-amino group of L,L-SDAP,[15] we synthesized L,L-SDAP derivatives with partially blocked free amino groups to prevent ninhydrin side reactions. *N*^6^-Methylated and *N*^6^-acetylated derivatives of L,L-SDAP were investigated and prioritized using molecular docking and modeling. Based on these data, a new enzymatic assay for *Hi*DapE is described using *N*^6^-methylated L,L-SDAP, which we have shown is simple and robust, and should be amenable to high-throughput screening. These data open the door to medicinal chemistry efforts toward the discovery of *Hi*DapE inhibitors that can function as a new class of antibiotics.

## Materials and methods

All solvents were distilled prior to use and all reagents were used without further purification unless otherwise noted. Ninhydrin was purchased as a 2% solution in 100% DMSO (dimethyl sulfoxide) with a lithium acetate buffer at pH 5.2. All synthetic reactions were conducted under an argon or nitrogen atmosphere, or as specified otherwise. The term reactor refers to a large round-bottom flask. Silica gel 60 Å, 40−75 μm (200 × 400 mesh), was used for column chromatography. Aluminum-backed silica gel 200 μm plates were used for TLC (thin-layer chromatography). ^1^H NMR spectra were obtained using a 300, 400, or 500 MHz spectrometer with trimethylsilane (TMS) as the internal standard. ^13^C NMR spectra were obtained using a 75, 100, or 125 MHz spectrometer. The purity of all compounds was determined to be ≥95% unless otherwise noted by high performance liquid chromatography (HPLC) employing a mobile phase A = 0.1% TFA in water and a mobile phase B = 0.1% TFA in acetonitrile with a gradient of 60% B increasing to 95% over 10 min, holding at 95% B for 5 min, then returning to 60% B and holding for 5 min. HRMS spectra were measured on a TOF instrument by electrospray ionization (ESI). HRMS spectra were collected using a Waters Acquity I class UPLC and Xevo G2-XS QTof mass spectrometer with a Waters Acquity BEH C18 column (1.7 μm, 2.1x50 mm). Mobile phase A was 0.05% formic acid in water and mobile phase B was 0.05% formic acid in acetonitrile. A gradient of 5% to 90% B over 5 min was applied. Enzyme activity curves were fitted with a program that utilizes non-linear least squares regression of the Gauss-Newton algorithm with optional damping using an ad hoc program.[16,17]

### Organic synthesis

#### (2*S*,6S)-2-(3-Carboxypropanamido)-6-(methylamino)heptanedioic acid hydrochloride (1b.HCl)

To a 100 mL reactor was added **10a** (1.50 g, 3.16 mmol) and a freshly prepared 6 M HCl solution (30 mL). The reaction mixture was allowed to stir at 25°C for 5 h and then concentrated under reduced pressure (0.1–10 mbar) at 25°C on a rotary evaporator and triturated in acetonitrile overnight at room temperature. The resulting slurry was filtered and dried under reduced pressure overnight to provide compound **1b** as the hydrochloride salt (**1b.HCl**, 1.04 g, 97%, deliquescent solid) as a white foam. ^1^H NMR (400 MHz, D_2_O): δ 4.35 (q, 1 H, *J* = 9.0, 5.0 Hz), 3.95 (t, 1 H, *J* = 6.2 Hz), 2,73 (s, 3 H), 2.64 (m, 2 H), 2.58 (m, 2 H), 2.03–1.88 (m, 3 H), 1.81–1.72 (m, 1 H), 1.58–1.46 (m, 1 H), 1.43–1.36 (m, 1 H). HRMS (IT-Tof): Calcd for C_12_H_21_N_2_O_7_ [M+H]^+^: 305.1349, Found: [M+H]^+^ 305.1309.

#### (2*S*,6*S*)-2-(3-Carboxylatopropanamido)-6-(methylamino)heptanedioate, 2,2,2-trifluoroacetate salt (1b.TFA)

To a solution of succinyl *t*-butyl ester **10b** (6.08 g, 14.8 mmol) in DCM (60 mL) was added a TFA/DCM solution (30/70, 135 mL) under nitrogen. The mixture was stirred at room temperature for 6 h and then concentrated on a rotary evaporator at 35–40°C. The resulting residue was dissolved in anhydrous methanol (6.0 mL) and slowly added to anhydrous diethyl ether (450 mL, HPLC grade, inhibitor-free) with vigorous stirring to precipitate the TFA salt. The slurry was allowed to stir overnight at room temperature. The slurry was filtered, and the wet cake was rinsed with anhydrous diethyl ether (50 mL), n-hexane (100 mL), and dried *in vacuo* at room temperature overnight to provide compound **1b** as the mono-methyl L,L-SDAP TFA salt (L,L-succinyl diaminopimelate trifluoroacetate; 6.06 g, 98% yield) as a white solid. ^1^H NMR (400 MHz, D_2_O): δ 4.36 (dd, 1H, *J* = 9.2, 5.2 Hz), 3.76 (t, 1H, *J* = 6.4 Hz), 2.72 (s, 3H), 2.69–2.66 (m, 2H), 2.66–2.59 (m, 2H), 1.98–1.92 (m, 3H), 1.80–1.77 (m, 1H), 1.52–1.50 (m, 1H), 1.44–1.42 (m, 1H). ^13^C NMR (100 MHz, D_2_O): δ 176.9, 175.6, 174.9, 172.3, 62.1, 52.3, 31.5, 30.0, 29.9, 29.0, 28.2, 20.5. HRMS (ESI-ToF): m/z calcd for C_12_H_21_N_2_O_7_^+^ [M+H]^+^: 305.1349, found: 305.1365. HPLC purity: 100%. Chiral HPLC: 100% e.e. Chiral HPLC was performed on a RegisPack column (250 x 4.6 mm, 5 micron) eluting with an isocratic gradient of hexane/isopropyl alcohol (90/10) + 0.1% TFA at 1.5 mL/min and monitoring at 210 nm.

#### (±)-(2*S*,6*S*)-2-Acetamido-6-(3-carboxylatopropanamido) heptanedioic acid (1c)

To a racemic mixture of *D*,*D*/*L*,*L*-SDAP (22.8 mg, 0.0786 mmol) was added glacial acetic acid (100 μL), acetic anhydride (17.3 μL, 0.236 mmol), and sodium acetate trihydrate (24 mg, 0.079 mmol). The reaction was degassed and agitated with stirring (200 rpm) at 30°C for 10 min, then after 1 hour at room temperature, reverse-phase TLC analysis eluting with H_2_O-AcOH-CH_3_CN (30-1-69) indicated complete consumption of the starting material. The resulting reaction mixture was quenched with 6 N HCl (0.1 mL) and concentrated to dryness under reduced pressure and the resulting oil was placed under high vacuum overnight where it became an off-white foam. The racemic product mixture was collected to afford the *N-*acetylated SDAP derivative **1c** as a colorless powder (38.5 mg, 100%). HPLC purity 90.3% as determined on a 5 micron Chirosil SCA(-) column (150 mm x 4.6 mm, 5 micron) with isocratic elution employing 84% MeOH in water containing 5 mM HClO_4_ with an elution time of 15 min with column temp at 25°C and a flow rate of 2 mL/min monitoring at a wavelength of 210 nm. ^1^H NMR (500 MHz, D_2_O): δ 4.68 (bs, ammonium ion), 4.07–4.02 (m, 2H), 2.55–2.43 (m, 4H), 1.92 (s, 3H), 1.75–1.65 (m, 2H), 1.63–1.53 (m, 2H), 1.29 (p, J = 8Hz, 2H). ^13^C NMR (500 MHz, D_2_O) δ 179.2, 179.1, 178.9, 174.9, 173.9, 55.1, 55.0, 31.4, 31.3, 31.0, 22.2, 22.1, 21.8. HRMS (IT-TOF) m/z calcd for C_13_H_20_N_2_O_8_ [M]^+^: 332.1232, found: 332.1220.

#### 1-(*tert*-Butyl) 5-methyl (*tert*-butoxycarbonyl)-L-glutamate (5)

According to the general method of Glinka,[18] to a 2 L reactor was added Boc-L-Glu-OtBu (**4**) (95.65 g, 315.3 mmol), milled K_2_CO_3_ (69.7 g, 504 mmol), and anhydrous DMAC (480 mL). To the reaction mixture was added methyl iodide (49.3 g, 21.2 mL, 112 mmol). The reaction was agitated with mechanical stirring (300 rpm) at 25°C for 18 h. To the reaction mixture was added methyl iodide (228 g, 100 mL, 1.61 mol) followed by Ag_2_O (110 g, 475 mmol). The resulting reaction mixture was stirred at 400 rpm with a water bath (15°C) in place to control the exotherm and allowed to further stir overnight (12 hours) and gradually warm to room temperature. ^1^H NMR analysis confirmed that the reaction had gone to completion. The reaction mixture was then diluted with EtOAc (750 mL) and treated with Celite^®^ (43 g), stirred for 15 min, filtered over a fritted funnel (fine porosity) containing a pad of Celite^®^. The filter was washed with EtOAc (500 mL). The filtrate was concentrated under reduced pressure at 40°C to remove EtOAc and DMAC to provide the crude product as an oil (168 g). The oil was diluted with EtOAc (500 mL) and heptane (400 mL) and washed with 50 wt% (weight %) sodium thiosulfate pentahydrate solution (2 x 500 mL), USP purified water (3 x 500 mL), brine (500 mL), dried with sodium sulfate (40 g)/silica gel (20 g), filtered, and concentrated to provide the title compound **5** (102 g, 97%) as a colorless oil. HPLC purity 97.7%. ^1^H NMR (400 MHz, CDCl_3_) δ 4.59 (m, amide rotamer, 0.50 H), 4.37 (m, amide rotamer, 0.50 H), 2.77 (d, mixture of amide rotamers, 3H), 2.38–2.20 (m, 3H), 2.00 (m, 1H), 1.45 (s, 18H). ^13^C NMR (100 MHz, CDCl_3_): δ 173.4, 173.3, 170.4, 170.2, 156.3, 155.8, 81.6, 81.5, 80.3, 80.0, 59.4, 58.3, 51.7, 31.3, 31.2, 30.9, 30.51, 28.4, 28.1, 24.3, 24.1. HRMS (ESI-ToF): m/z calcd for C_16_H_29_NNaO_6_^+^ [M+Na]^+^: 354.1887, found: 354.1920.

#### *tert*-Butyl-(*S*)-2-((tert-butoxycarbonyl)(methyl)amino)-5-hydroxypentanoate (6)

To a 3 L 3-necked reactor was added methyl ester **5** (100.0 g, 301.7 mmol) and ethanol (HPLC grade, 1500 mL) and the mixture was cooled to < 5°C in an ice water bath. Sodium borohydride (57.6 g, 1520 mmol) was added in 5 portions over 15 min. The reaction was allowed to stir at < 5°C and subsequently allowed to gradually warm to room temperature overnight while the cooling bath remained in place. After 20 hours, TLC analysis (EtOAc/heptane, 40/60) showed complete consumption of the methyl ester starting material (Rf = 0.51) and the presence of the alcohol product spot (Rf = 0.22). To a 12 L reactor containing a cold solution of 50% aqueous ammonium chloride solution (3000 mL, < 5°C) was slowly added the reaction mixture over a period of 15 min such that the temperature remained < 15°C. The mixture was allowed to stir at 15–20°C for 30 min, then EtOAc (1000 mL) was added. The organic layer was separated, and the aqueous layer was further extracted with EtOAc (3 x 1000 mL). The combined organic layers were washed with brine (300 mL), dried with sodium sulfate, filtered, and concentrated to a colorless oil (90.8 g). The crude oil was dissolved in EtOAc/heptane (20/80, 100 mL), which was purified through a silica gel plug (1000 g) and the plug was flushed with EtOAc/heptane (20/80, 2 L) and EtOAc/heptane (40/60, 6 L) to afford the title alcohol **6** (88.6 g, 97%) as a colorless oil. ^1^H NMR: (400 MHz, CDCl_3_): δ 4.65 (m, amide rotamer, 0.50 H), 4.35 (m, amide rotamer, 0.50 H), 3.68 (t, 2H, *J* = 6.4 Hz), 2.78 (m, mixture of amide rotamers, 3H), 2.04–1.71 (m, 3H), 1.69–1.53 (m, 2H), 1.45 (s, 18H). ^13^C NMR (100 MHz, CDCl_3_): δ 171.1, 171.0, 156.6, 156.0, 81.4, 81.4, 80.2, 78.0, 62.2, 62.1, 59.7, 58.4, 30.6, 29.4, 29.2, 28.4, 2819, 25.5, 25.3. MS M+1 (304 m/z), M+23 (326 m/z), M+39 (342 m/z), and 2M+23 (629 m/z). HRMS (ESI-ToF): m/z calcd for C_15_H_29_NNaO_5_^+^ [M+Na]^+^: 326.1938, found: 326.1969.

#### *tert*-Butyl (*S*)-2-((tert-butoxycarbonyl)(methyl)amino)-5-oxopentanoate (7)

To a 250 mL reactor containing alcohol **6** (6.09 g, 20.1 mmol) was added DCM (90 mL) followed sequentially by PCC (pyridinium chlorochromate, 6.50 g, 44.1 mmol) and silica gel (60–200 micron, 7.00 g) with DCM (18 mL) as a rinse. The mixture was allowed to stir at room temperature for 1 hour after which time TLC analysis (EtOAc/heptane, 40/60) showed complete consumption of starting material and the presence of aldehyde product spot (Rf = 0.48). The reaction mixture was filtered through a plug of silica gel (60–200 micron, 90 g) packed in heptane, and product was eluted off the silica plug sequentially with DCM (350 mL), 20% EA/heptane (500 mL), 40% EA/heptane (500 mL), and 100% EA (250 mL) to afford aldehyde **7** (5.67 g, 94%) as a colorless oil. ^1^H NMR (400 MHz, CDCl_3_): δ 9.78 (d, 1H, amide rotamer), 4.59 (m, amide rotamer, 0.50 H), 4.34 (m, amide rotamer, 0.50 H), 2.77 (d, mixture of amide rotamers, 3H), 2.51–2.40 (m, 2H), 2.28–2.22 (m, 1H), 1.98 (m, 1H), 1.46 (s, 18H). ^13^C NMR (100 MHz, CDCl_3_): δ 201.4, 201.1, 177.7, 170.3, 170.2, 156.5, 155.9, 81.8, 80.6, 80.3, 59.4, 59.4, 58.6, 58.4, 40.8, 40.4, 31.4, 31.3, 30.8, 30.4, 28.5, 28.1, 24.1, 24.0, 21.6, 21.5. HRMS (ESI-ToF): m/z calcd for C_15_H_27_NNaO_5_^+^ [M+Na]^+^: 324.1781, found: 324.1816.

#### (*S*,*Z*)-7-*tert*-Butyl 1-methyl 2-(benzyloxycarbonylamino)-6-(*tert*-butoxycarbonyl(methyl)amino)hept-2-enedioate (8a)

In a 250 mL round bottomed reactor was placed, under an argon atmosphere, (±)-Cbz-α-phosphonoglycine trimethyl ester **11** (3.96 g, 11.9 mmol) and DCM (30 mL), then DBU (1.71 g, 11.2 mmol) was added followed by a DCM rinse (7 mL). The mixture was allowed to stir at room temperature for 45 min and subsequently added to a solution of aldehyde **7** (3.00 g, 9.93 mmol) in DCM (40 mL) over 2 min to give a pale-yellow solution. The reaction was allowed to stir for 2 hours and concentrated under reduced pressure at 40–45°C. The residue was dissolved in isopropyl acetate (100 mL) and was washed with 6% citric acid (3 x 100 mL), USP purified water (75 mL), brine (75 mL), dried with sodium sulfate for 30 min, filtered, and concentrated to give a crude oil. The crude oil (dissolved in EtOAc/heptane, 20/80) was purified through a glass gravity column (10 in x 2.75 in) packed with silica gel (Silicycle, 60–200 micron, 125 g) in heptane. The column was eluted with EtOAc/heptane gradient (10/90 to 40/60). Appropriate fractions containing pure product were combined and concentrated to afford the enamide **8a** (4.55 g, 90% yield, viscous colorless oil). HPLC purity 96.4%. ^1^H NMR (400 MHz, CDCl_3_): δ 7.36–7.31 (m, 5 H), 6.76 (bs, amide rotamer, 0.70 H), 6.59 (m, 1 H), 6.28 (bs, 0.30 H), 5.12 (m, 2 H), 4.55 (m, 0.60 H), 4.30 (m, 0.40 H), 3.76 (bs, 3 H), 2.77–2.71 (s, 3 H, amide rotamers), 2.23 (m, 2 H), 2.01 (m, 1 H), 1.86 (m, 1 H), 1.45 (bs, 18 H). HRMS (ESI-ToF): m/z calcd for C_26_H_38_N_2_NaO_8_ [M+Na]^+^: 529.2520, found: 529.2553.

#### 1-Benzyl 7-(*tert*-butyl) (*S*,*Z*)-2-(((benzyloxy)carbonyl)amino)-6-((*tert*-butoxycarbonyl)(methyl)amino)hept-2-enedioate (8b)

To a 500 mL reactor was added benzyl *Z*-phosphinoglycine dimethyl ester **13** (22.00 g, 54.00 mmol) and DCM (100 mL). At ambient temperature, DBU (7.88 g, 51.8 mmol) was added to the mixture under argon. After 30 min, a solution of aldehyde **7** (14.15 g, 84% pure based on ^1^H NMR, 39.44 mmol) in DCM (150 mL) was slowly added via dropping funnel over 10 min. The reaction mixture was allowed to stir at room temperature for 21 h and subsequently was concentrated, diluted with ethyl acetate (350 mL), and washed with USP purified water (175 mL). The aqueous stream was extracted with ethyl acetate (2 x 85 mL). The combined organic streams were successively washed with 6% citric acid (110 mL), saturated aqueous sodium bicarbonate (110 mL), brine (110 mL), dried with sodium sulfate and concentrated to provide the crude enamide product (30.7 g) as a yellow oil. The crude product dissolved in diethyl ether (350 mL) and silica gel (48 g, 60–200 micron) were combined and concentrated to dryness to afford a dry-loaded material that was placed in a 750 g cartridge coupled to another cartridge containing silica gel (615 g, 60–200 micron) equilibrated with heptane. Purification was performed on a Teledyne Isco Rf Flash chromatography unit eluting with a step-gradient of 100% heptane (2 CV), 2% IPA (isopropyl alcohol) in heptane (5 CV), and 5% IPA in heptane (4.5 CV) at 200 mL/min providing pure enamide Horner-Emmons product **8b** (21.4 g, 93%) as a colorless viscous oil. ^1^H NMR (400 MHz, CDCl_3_): δ 7.34 (bm, 10H), 6.77 (bs, amide rotamer, 0.60H), 6.62 (m, 1H), 6.29 (bs, amide rotamer, 0.40H), 5.19 (s, 2H), 5.11 (d, amide rotamer, 2H), 4.55 (m, amide rotamer, 0.60 H), 4.29 (m, amide rotamer, 0.40 H), 2.73 (d, mixture of amide rotamer, 3H), 2.24 (m, 2H), 2.02 (m, 1H), 1.98 (m, 1H), 1.85 (m, 1H), 1.43 (s, 18H). ^13^C NMR (100 MHz, CDCl_3_): δ 170.5, 164.5, 164.4, 156.9, 155.8, 154.6, 154.1, 136.7, 136.2, 136.0, 135.7, 135.5, 128.6, 128.6, 128.5, 128.3, 128.2, 81.7, 80.4, 80.4, 67.5, 67.4, 67.3, 67.2, 59.6, 58.2, 30.7, 28.4, 28.1. HRMS (ESI-ToF): m/z calcd for C_32_H_42_N_2_NaO_8_^+^ [M+Na]^+^: 605.2833, found: 605.2928.

#### (2*S*,6*S*)-7-*tert*-Butyl 1-methyl 2-(benzyloxycarbonylamino)-6-(*tert* butoxycarbonyl(methyl)amino)heptanedioate (9a)

To a Mettler Toledo RC1e system equipped with a MP06-HC 1.2-liter pressure glass reactor, mechanical stirrer, and an external temperature control unit under nitrogen was added a solution of enamide **8a** (4.00 g, 7.90 mmol) in HPLC-grade methanol (110 mL, degassed with argon). A solution of Rh(I)(COD)(*S*,*S*)-Et-DuPHOS catalyst (86 mg, 0.12 mmol) in degassed methanol (HPLC grade, 10 mL) was added and the reactor was pressurized with nitrogen (3x) and hydrogen (3x), then placed under 50 psi of hydrogen. The mixture was agitated with mechanical stirring (500 rpm) at 25°C for 25 h after which ^1^H NMR indicated complete consumption of the alkene. The reaction mixture was purged with nitrogen three times and then concentrated under reduced pressure. The crude residue (4.04 g) was dissolved in ethyl acetate/heptane (50/50, 8 mL) and passed through a silica gel plug (20.5 g) eluting with ethyl acetate/heptane (50/50, 200 mL) to afford Cbz-protected amino acid derivative **9a** (3.74 g, 93% yield) as a colorless viscous syrup. HPLC purity (achiral): 100% AUC (area under the curve). Chiral HPLC: e.e. = 97.4%. Chiral HPLC was performed using a Chiralpak AD-H column (250 x 4.6 mm, 5 micron) eluting with a gradient of hexane/EtOH (90/10 to 30/70) over 20 min at 1.0 mL/min and monitoring at 210 nm. ^1^H NMR (400 MHz, CDCl_3_): δ 7.36–7.30 (m, 5 H), 5.30–5.10 (s, 3 H, amide rotamers), 4.60 (m, 0.55 H), 4.34 (m, 1.45 H, amide rotamer), 3.74–3.68 (s, 3 H, amide rotamers), 2,76–2.71 (s, 3 H, amide rotamers), 1.83 (m, 2 H), 1.74 (m, 2 H), 1.44–1.26 (m, 20 H). ^13^C NMR (100 MHz, CDCl_3_): δ 172.9, 172.8, 170.9, 170.7, 156.5, 155.99, 155.8, 136.2, 128.5, 128.1, 81.4, 81.3, 80.1, 79.9, 67.0, 59.4, 57.9, 53.7, 53.6, 52.4, 32.1, 31.9, 30.5, 28.4, 28.0, 21.8. HRMS (ESI-ToF): m/z calcd for C_26_H_40_N_2_NaO_8_^+^ [M+Na]^+^: 531.2677, found: 531.2726.

#### 1-Benzyl-7-(*tert*-butyl) (2*S*,6*S*)-2-(((benzyloxy)carbonyl)amino)-6-((*tert*-butoxycarbonyl)(methyl)amino)heptanedioate (9b)

To a Mettler Toledo RC1e system equipped with a MP06-HC 1.2-liter pressure glass reactor, mechanical stirrer, and an external temperature control unit under nitrogen was added a solution of enamide **8b** (19.9 g, 34.1 mmol) in HPLC grade methanol (110 mL, degassed with argon). This mixture was sparged with argon for an additional 30 min. A solution of Rh(I)(COD)(*S*,*S*)-Et-DuPHOS catalyst (378 mg, 0.52 mmol) in degassed methanol (HPLC grade, 50 mL) was added and the reactor was pressurized sequentially with nitrogen (3x), hydrogen (3x), and finally placed under 50 psi of hydrogen. The mixture was agitated with mechanical stirring (500 rpm) at room temperature for 21 hours after which ^1^H NMR indicated complete consumption of the alkene. The reaction mixture purged with nitrogen three times, then concentrated under reduced pressure. The crude residue (19.7 g) was dissolved in ethyl acetate/heptane (50/50, 45 mL) and passed through a silica gel plug (106 g) using ethyl acetate/heptane (50/50, 600 mL) to afford Cbz-protected amino acid derivative **9b** (19.3 g, 97% yield) as a colorless viscous oil. HPLC analysis (achiral): 95.1%. Chiral HPLC: e.e. = 92%. Chiral HPLC was performed using a RegisPack column (250 x 4.6 mm, 5 micron) eluting with an isocratic gradient for 15 min consisting of hexane/IPA (70/30) + 0.1% DEA at 1.5 mL/min and monitoring at 220 nm. ^1^H NMR (400 MHz, CDCl_3_): δ 7.35 (m, 10 H), 5.28 (t, 0.89 H, *J* = 8.0 Hz, amide rotamer), 5.17–5.10 (m, amide rotamer, 4.10 H), 4.56 (m, 0.53 H, amide rotamer), 4.41 (m, 0.90 H, amide rotamer), 4.23 (m, 0.58 H, amide rotamer), 2.71 (d, mixture of amide rotamers, 3H), 1.89–1.59 (m, 4 H), 1.50–1.25 (m, 20 H). ^13^C NMR (100 MHz, CDCl_3_): δ 172.4, 172.3, 170.9, 170.8, 156.6, 156.1, 156.0, 155.9, 136.3, 135.4, 135.3, 128.7, 128.6, 128.5, 128.4, 128.3, 128.2, 81.5, 81.4, 80.2, 80.0, 67.3, 67.3, 67.1, 59.6, 58.1, 54.0, 53.8, 32.2, 32.0, 30.6, 28.6, 28.5, 28.1, 22.02. HRMS (ESI-ToF): m/z calcd for C_32_H_44_N_2_NaO_8_^+^ [M+Na]^+^: 607.2990, found: 607.3088.

#### 4-((2*S*,6*S*)-7-*tert*-Butoxy-6-(*tert*-butoxycarbonyl(methyl)amino)-1-methoxy-1,7-dioxoheptan-2-ylamino)-4-oxobutanoic acid (10a)

To a 250 mL reactor under argon was added a solution of **9a** (3.15 g, 6.19 mmol) in HPLC grade methanol (85 mL). The mixture was sparged with argon for 10 min followed by addition of 10% Pd/C (320 mg). The reactor was flushed with argon for 5 min, hydrogen for 5 min, then placed under balloon pressure of hydrogen and allowed to stir overnight (16 h) at room temperature after which time TLC analysis (EtOAc/DCM/MeOH, 20/75/5) showed complete consumption of starting material. The mixture was sparged with argon, filtered over a pad of Celite^®^, rinsed with methanol (50 mL) and the resulting filtrate was concentrated and chased with toluene to provide the free deprotected amine (2.34 g, 100%) as a colorless oil. ^1^H NMR (400 MHz, CDCl_3_): δ 4.60 (m, 0.50 H, amide rotamer), 4.32 (m, 0.50 H, amide rotamer), 3.72 (s, 3H), 3.47 (m, 1H), 2.77 (s, 3H, amide rotamer), 1.90–1.60 (m, 6H), 1.47 (m, 20H). ^13^C NMR (100 MHz, CDCl_3_): δ 176.5, 171.2, 171.0, 156.5, 156.0, 81.4, 81.3, 80.2, 79.9, 59.6, 58.3, 54.4, 54.3, 52.1, 34.4, 34.4, 30.7, 28.8, 28.5, 28.1, 22.4. HRMS (ESI-ToF): m/z calcd for [M+H]^+^ for C_18_H_35_N_2_O_6_^+^: 375.2490, found: 375.2514.

To a 250 mL reactor was added the Cbz-deprotected amine (2.25 g, 6.01 mmol), succinic anhydride (0.66 g, 6.6 mmol), and DCM (50 mL). The mixture was cooled to < 5°C and triethylamine (0.92 mL, 6.6 mmol) was added dropwise over 2 min. The cooling bath was removed, and the reaction was allowed to stir overnight at room temperature. After 24 hours, the reaction mixture was diluted with DCM (50 mL), washed sequentially with 6% citric acid (2 x 50 mL), brine (25 mL), dried with sodium sulfate, filtered, and concentrated under reduced pressure and chased with DCM and heptane to furnish **10a** (2.90 g, 99%) as a colorless viscous syrup, which was used without further purification. ^1^H NMR (400 MHz, CDCl_3_): δ 6.43 (d, 0.50H, *J* = 8.0 Hz, amide rotamer), 6.32 (d, 0.50H, *J* = 8.0 Hz, amide rotamer), 4.69–4.57 (m, 1.5H, amide rotamer), 4.36–4.32 (m, 0.50H, amide rotamer), 3.75 (d, 3H, mixture of amide rotamers), 2.83–2.72 (m, 4H), 2.65–2.42 (m, 3H), 1.88–1.70 (m, 5H), 1.46–1.44 (m, 19H). HRMS (ESI-ToF): m/z calcd for C_22_H_38_N_2_NaO_9_^+^ [M+Na]^+^: 497.2470, found: 497.2493.

#### 4-(((2*S*,6*S*)-1-(benzyloxy)-7-(*tert*-butoxy)-6-((*tert*-butoxycarbonyl)(methyl)amino)-1,7-dioxoheptan-2-yl)amino)-4-oxobutanoic acid (10b)

To a 500 mL reactor was added Cbz-protected amino acid **9b** (10.6 g, 18.2 mmol) and methanol (275 mL). The mixture was sparged with nitrogen for 10 min followed by addition of 10% Pd/C (1.04 g). The reactor was flushed with nitrogen for 5 min, hydrogen for 5 min, then placed under balloon pressure of hydrogen for 6 h. The mixture was treated with Celite^®^ (5.0 grams) and sparged with nitrogen for 10 min, filtered over a pad of Celite^®^, and rinsed with methanol (200 mL). The resulting filtrate was concentrated to provide the free amino acid (6.39 g, 97% yield) as an off-white solid. HRMS of the free amino acid (ESI-ToF): m/z calcd [M+H]^+^ for C_17_H_33_N_2_O_6_^+^: 361.2333, found: 361.2377.

To a 500 mL reactor was added the free amino acid (6.02 g, 16.7 mmol), succinic anhydride (1.73 g, 17.3 mmol), and DCM (175 mL). The mixture cooled to < 5°C and triethylamine (3.66 g, 36.2 mmol) was added dropwise over 3 min. The cooling bath was removed, and the reaction was allowed to stir for 2 h at room temperature. HPLC/UPLC/TLC analysis showed that the reaction was complete. The reaction mixture was concentrated, diluted with EtOAc (300 mL), washed sequentially with 0.10 N HCl (390 mL), USP purified water, (250 mL), brine (200 mL), dried with sodium sulfate, filtered, and concentrated to provide crude **10b** (7.40 g). The crude product **10b** was dissolved in EtOAc (25 mL) and was purified by passing through a silica gel plug (52 g, 60–200 micron, equilibrated with EtOAc/THF (1:1) followed by 100% EtOAc). The silica plug was flushed with EtOAc (100 mL) and EtOAc/THF (1:1, 250 mL). Appropriate fractions were combined, concentrated, and chased with heptane affording pure succinate adduct **10b** (7.10 g, 92% yield). HPLC purity: 98.6% AUC. Chiral HPLC: e.e. = 100%. Chiral HPLC was performed using a RegisPack column (250 x 4.6 mm, 5 micron) eluting with an isocratic eluent for 15 min consisting of hexane/IPA (90/10) + 0.1% TFA at 1.5 mL/min and monitoring at 210 nm. ^1^H NMR (400 MHz, CDCl_3_): δ 10.74 (bs, 2H), 7.10 (d, 1H, *J* = 8.0 Hz), 4.65–4.51 (m, amide rotamer, 1.5H), 4.29–4.27 (m, amide rotamer, 0.5H), 2.78–2.49 (m, mixture of amide rotamers, 7H), 1.87–1.60 (m, 4H), 1.45 (d, 18H), 1.38–1.16 (m, 2H). ^13^C NMR (100 MHz, CDCl_3_): δ 176.5, 176.5, 175.5, 175.4, 173.1, 172.9, 170.8, 157.1, 156.8, 81.7, 81.0, 80.8, 67.1, 59.9, 58.4, 53.5, 52.5, 52.1, 31.3, 30.7, 30.6, 30.4, 29.7, 28.5, 28.3, 28.1, 22.3, 21.7. HRMS (ESI-ToF): m/z calcd for C_21_H_36_N_2_NaO_9_ [M+Na]^+^: 483.2313, found: 483.2368.

#### (±)-Cbz-α-phosphonoglycine dimethyl ester (12)

According to a reported patent procedure,[19] to a 1 L 3-neck reactor equipped with a mechanical stirrer, J-Kem thermocouple, nitrogen inlet, and water bath (20–25°C), was added (±)-Cbz-α-phosphonoglycine trimethyl ester **11** (33.1 g, 100 mmol), MeOH (180 mL), and USP purified water (20 mL). To the slightly hazy mixture was added a solution of 1 N aq NaOH (102 mL, 102 mmol) *via* dropping funnel over a period of 1 h to give a homogeneous mixture. The resulting mixture was allowed to stir for an additional 1 h at room temperature and subsequently concentrated under reduced pressure at 25–30°C to remove methanol. The aqueous residue was polished filtered through filter paper and pH adjusted to pH 1.5 with 1 N H_2_SO_4_ solution (100 mL) to provide a white murky solution. The mixture was extracted with EtOAc (4 x 150 mL) and the combined organic streams were washed with brine (150 mL), dried with magnesium sulfate, and concentrated under reduced pressure to afford (±)-Cbz-α-phosphonoglycine dimethyl ester **12** (31.0 g, 98%) as a viscous oil that solidified to a white solid upon standing. HPLC purity 97.4%. The proton NMR data are in accordance with that reported in the literature.[20] HRMS (ESI-ToF): m/z calcd for C_12_H_17_NO_7_P^+^ [M+H]^+^: 318.0737, found: 318.0782.

#### Benzyl 2-(benzyloxycarbonylamino)-2-(dimethoxyphosphoryl)acetate (13)

According to the general procedure of Nakanishi,[21] a 500 mL 3-neck reactor was charged (±)-Cbz-α-phosphonoglycine dimethyl ester **12** (30.8 g, 97.1 mmol), ACN (80 mL), and DBU (15.2 g, 21.0 mL, 99.8 mmol) to provide a clear solution. The mixture was allowed to stir for 10 min at room temperature with a cooling bath in place (ambient temperature). To the reaction mixture was added benzyl bromide (17.4 g. 102 mmol) dropwise over 5 min. After 2 hours at room temperature, the reaction mixture was concentrated under reduced pressure and diluted with EtOAc (350 mL). The organic mixture was washed with USP purified water (130 mL), 3% citric acid solution (1 x 110 mL), saturated NaHCO_3_ solution (100 mL), brine (100 mL), dried with sodium sulfate, and concentrated under reduced pressure to give the crude product (43.5 g). The crude product was dissolved in EtOAc/heptane (1:1, 90 mL) at 45°C, seeded, and diluted with heptane (350 mL) and the resulting slurry was allowed to stir an additional 1 h at ambient temperature, and filtered. The filter cake was washed with heptane (50 mL) and pulled dry to give benzyl 2-(benzyloxycarbonylamino)-2-(dimethoxyphosphoryl)acetate **13**[22] (38.71 g, 98%) as a white solid. HPLC purity: 95.9% AUC. ^1^H NMR: (400 MHz, CDCl_3_): δ 7.35 (m, 10H), 5.62 (d, 1H, *J* = 12.0 Hz), 5.29 (d, 1H, *J* = 12.0 Hz), 5.22 (d, 1H, *J* = 12.0 Hz), 5.14 (dd, 2H, *J* = 12.0, 4.0 Hz), 4.96 (dd, 24.0, 12.0 Hz), 3.72 (d, 6H. *J* = 12.0 Hz). HRMS (ESI-ToF): m/z calcd for C_19_H_23_NO_7_P^+^ [M+H]^+^: 408.1207, found: 408.1255.

### *Hi*DapE molecular modeling

Models of L,L-SDAP (**1a**), *N*^6^-methyl-L,L-SDAP (**1b**), and *N*^6^-acetyl-L,L-SDAP (**1c**) bound to *Hi*DapE were developed using the Molecular Operating Environment (MOE)[23] computational suite’s Builder utility followed by minimization in the gas phase using the force field MMFF94X. The minimized ligands were then subjected to the Conformational Search protocol to generate structural-conformation databases populated with as many as 10,000 individual conformations. Conformational databases were generated for all three ligands of interest for use in the following docking experiments.

The previously-reported X-ray crystal structure of the product-bound form of *Hi*DapE in the closed conformation (PDB: 5VO3)[24] were uploaded into MOE. Following receptor preparation, molecular docking was performed using the previously-generated ligand conformation databases. Substrate analog docking was carried out in the prepared *Hi*DapE enzyme model with the products and solvent atoms inactivated and the docking site specified at the catalytic Zn(II) atoms. Substrate analog placement employed the Proxy Triangle method with London dG scoring generating 50 data points that were further refined using the induced fit method with GBVI/WSA dG scoring to obtain the top 30 docking results. The docking protocol was repeated for all three substrates and analyzed for selection of the best docking pose. Docking poses of the substrate **1a** and analogs **1b** and **1c** were assessed as judged by their similarity to the product binding interactions seen in the original product-bound X-ray crystal structure. A single substrate analog docking pose was selected for each of the three substrates providing initial substrate-bound enzyme models. The *N*^6^,*N*^6^-dimethyl-L,L–SDAP analog was initially pursued in parallel with the monomethyl analog **1b**, but was more problematic synthetically, and was ultimately set aside based on modeling that suggested it would be a very poor substrate (S1 Fig).

The three substrate/enzyme models were then solvated in a simple water box at pH of 7.4 that was treated with NaCl counterions to balance the charge. Periodic boundary conditions were enabled, and the hydrogen bonding network of the model was optimized by automatically sampling different tautomer/protomer states using Protonate3D,[25] which calculates optimal protonation states, including titration, rotamer, and “flips” using a large-scale combinatorial search.[26] The system atoms were then optimized with a short, localized molecular minimization process with atoms further then 8 Å from the substrate fixed. System refinement continued until an RMS Gradient of 0.1 kcal/mol/Å was attained. Molecular Dynamics parameters were set to globally minimize the protein, substrate, and solvent atoms in the system using an NPA algorithm with an Amber12:EHT force field, with a typical heating and cooling protocol. Simulation results were then minimized once again before the final binding poses were obtained for comparison.

### Protein expression, purification, and Zn(II) binding

Recombinant *Hi*DapE was expressed and purified according to a published protocol.[11] Briefly, several grams of cell paste were thawed at room temperature and lysed by sonication using 2 s pulses with 5 s rest periods. The cell debris was removed by centrifugation at 4°C for 40 min at 12,000 g. The supernatant was applied to a column packed with 10 mL of HisTrap HP resin (GE Healthcare), connected to VacMan (Promega) and the chromatographic process was accelerated with a vacuum pump. The column was washed with 20 bed volumes of lysis buffer and the His_6_-tagged *Hi*DapE enzyme was eluted with 25 mL of elution buffer (50 mM HEPES (4-(2-hydroxyethyl)-1-piperazineethanesulfonic acid) pH 8.0, 500 mM NaCl; 500 mM imidazole; 2 mM DTT). The His_6_-tag was cleaved with TEV protease (2 mg of a His_6_-tagged form) overnight at 4°C and dialyzed to remove the excess imidazole. The resulting solution was mixed with His-Trap HP resin to capture the cleaved His_6_-tag and the His_6_-tagg TEV protease. The flow-through containing *Hi*DapE was collected, concentrated, and loaded on an SEC column and the eluent was concentrated using an Amicon YM-10 membrane to a concentration 20 mg/ml^-1^ followed by the addition of the reducing agent TCEP (1 mM). Purified *Hi*DapE exhibited a single band on SDS-PAGE (41,500 Da). Protein concentrations were determined from the absorbance at 280 nm using the previously reported molar absorptivity that was calculated using the method developed by Gill and Hippel.[27] The protein concentration determined using this molar absorptivity was in close agreement to that obtained using a Bradford assay. Individual aliquots of purified *Hi*DapE were stored in liquid nitrogen until needed.

Apo-*Hi*DapE was prepared by extensive dialysis for 3 to 4 days against 10 mM EDTA in 50 mM HEPES buffer, pH 7.5. The enzyme was then exhaustively dialyzed against metal-free (Chelexed) 50 mM HEPES buffer, pH 7.5. Apo-*Hi*DapE samples were incubated with 1.7 equivalents of ZnCl_2_ (97%; Sigma-Aldrich) in 50 mM HEPES buffer for 30 min.

### Enzyme assay studies

#### Modified L,L-SDAP as potential substrates

The hydrolytic activity of *Hi*DapE toward alternate substrates **1b** and **1c** was monitored by the decrease in absorbance at 225 nm using a Shimadzu UV-2450 UV/Visible Spectrophotometer. All reaction volumes were 1 mL with 8 nM *Hi*DapE, 5 mM **1b** or 5 mM **1c** in 50 mM HEPES buffer, pH 7.5. Spectra were recorded over 35 min with one scan per minute. Enzyme dilutions were made directly before each trial from a concentrated stock solution that was stored at -80°C.

#### *Hi*DapE assay protocol

All reaction volumes were 200 μL with 25 nM *Hi*DapE and 1 mM *N*^6^-methyl-L,L-SDAP **1b** unless otherwise stated. Ninhydrin was purchased as a 2% solution in 100% DMSO/lithium acetate buffer at pH 5.2. Initial assay conditions used to measure the activity of *Hi*DapE were carried out in triplicate as follows: to a 50 mM HEPES buffered *Hi*DapE solution at pH 7.5 at room temperature (18–22°C), **1b** as the TFA salt was added. The reaction was allowed to proceed for 10 min after which 100 μL of the 2% ninhydrin solution was added. The reaction mixture was vortexed and heated to 80°C for 15 min followed by cooling on ice for 2 min. The absorbance at 570 nm was recorded on a BioTek Syngen microplate reader.

#### Ninhydrin reactions with glutamic acid and sarcosine

Control reactions were carried out using glutamic acid as a primary amine model and sarcosine as a model secondary amine. UV/Vis absorbances between 300 and 800 nm were recorded on a Shimadzu UV-2450 UV/Visible Spectrophotometer and the time course development was followed by reading the absorbance at 570 nm using a BioTek Syngen microplate reader. Control reactions for the detection of primary amines were carried out in triplicate as follows: to a 50 mM HEPES pH 7.5 buffered solution at 30°C was added glutamic acid (0–1.2 mM) and 100 μL of the 2% ninhydrin solution. The reaction mixture was heated to 100°C (2 min vs 15 min), cooled on ice and the absorption spectra recorded. Control reactions for the detection of secondary amine were carried out in triplicate in the same manner except substituting with the secondary amine sarcosine.

Time course plots of glutamic acid or sarcosine with ninhydrin development were carried out in triplicate as follows: to a 50 mM HEPES buffered solution pH 7.5 was added 1 mM amine at 30°C. After a 5 min incubation period, a 2% ninhydrin solution was added and the reaction vortexed followed by heating at 100°C for time intervals of 0, 2, 3, 5, 10, 15 and 20 min. The mixtures were cooled on ice for 2 min and 80μL of each reaction was examined via a BioTek Syngen microplate reader at 570 nm. These data were also obtained at 80°C and 60°C (S2 and S3 Figs).

#### Incubation of *Hi*DapE with DMSO

All reaction volumes were 200 μL containing 25 nM *Hi*DapE, 1 mM **1b** as the TFA salt, and the 2% ninhydrin solution in DMSO. UV/Vis spectra were recorded on 80 μL aliquots of ninhydrin-developed reactions unless otherwise stated, and absorption data were collected on a BioTek Syngen microplate reader. Control reactions were carried out in triplicate as follows: to a 50 mM HEPES, pH 7.5 buffered solution of *Hi*DapE at 30°C was added 1 mM **1b**. The reaction was allowed to proceed for 10 min after which 100 μL of the 2% ninhydrin solution was added and subsequently heated to 80°C for 15 min. This reaction was quenched by placed on ice until cooled to 30°C. The absorbance was recorded at 570 nm on a BioTek Syngen microplate reader. These control reactions were set as 100% *Hi*DapE activity.

The DMSO incubation reactions were carried out in triplicate as follows: to a 50 mM HEPES, pH 7.5 buffered *Hi*DapE solution at 30°C was added DMSO (75% v/v). *Hi*DapE was incubated for 0 to 10 min after which 1mM **1b** was added and the reaction was allowed to proceed for 10 min. The enzymatic reaction was then quenched by the addition of the 2% ninhydrin solution. The reaction mixture was vortexed and heated to 80°C for 15 min followed by cooling on ice until the solution was 30°C. The absorbance was recorded at 570 nm on a BioTek Syngen microplate reader.

#### Monitoring thermal denaturation of *Hi*DapE with circular dichroism (CD)

Denaturation of *Hi*DapE was determined using an Olis DSM 20 circular dichroism spectrophotometer. Samples were measured in a cylindrical quartz cuvette with a 1 mm path length, and contained 10 mM phosphate, pH 8.0, and 0.4 μM of *Hi*DapE. Data was collected every 1 nm over a wavelength range of 190–260 nm. The temperature was increased from 20°C to 80°C using 10°C increments with 2 min incubation times at each temperature (S4 Fig). At 80°C, the temperature was held constant until complete denaturation was observed. OlisGlobalworks software v1.3 was used to deconvolute the spectra and calculate the percent alpha helices and beta sheets.

#### Pre-heat incubation control reactions

All reaction volumes were 200 μL containing 25 nM *Hi*DapE, 1 mM **1b** as the TFA salt, and the 2% ninhydrin solution in DMSO. UV/Vis spectra were recorded on 80 μL aliquots of ninhydrin-developed reactions unless otherwise stated, and absorption data were collected on a BioTek Syngen microplate reader. The control reactions were carried out in triplicate under the previous conditions used for 100% standard enzymatic activity of *Hi*DapE. A second set of control reactions using denatured *Hi*DapE, to establish zero activity, were obtained in triplicate by heating *Hi*DapE to 100 ^o^C for 3 min as follows: a 50 mM HEPES, pH 7.5 buffered *Hi*DapE solution was heated at 100°C from 1 to 5 min followed by cooling on ice to 30°C. To this solution was added 1 mM **1b** and the reaction was allowed to proceed for 10 min after which a 2% ninhydrin solution was added. The reaction mixture was vortexed and heated at 80°C for 15 min. The ninhydrin reaction was quenched by placing in ice and the absorbance was recorded at 570 nm (S5 Fig).

#### Optimizing DapE enzyme concentration for the assay

Reactions varying the DapE enzyme concentration were carried out as follows: to a solution of 50 mM HEPES, pH 7.5 was added the desired concentration of enzyme at 30°C followed by the addition of 20 μL of **1b** as the TFA salt (final concentration of 1 mM in 200 μL total volume). The reaction was allowed to proceed for 10 min after which the enzymatic activity was quenched by heating to 100°C for 1 min and cooled on ice. Next, 100 μL of a 2% ninhydrin solution was added to a final concentration of 300 μL. The reaction was heated to 80°C for 15 min followed by cooling on ice until the solution was 30°C. The absorbance was recorded at 570 nm on a BioTek Syngen microplate reader.

#### Ninhydrin-based assay for *Hi*DapE enzymatic activity

The protocol for the ninhydrin-based assay to determine enzymatic activity of *Hi*DapE was determined in triplicate as follows: to a 50 mM HEPES, pH 7.5 buffered solution containing 8 nM *Hi*DapE at 30°C was added 2 mM **1b** (final volume 200 μL). The reaction was allowed to proceed for 10 min and then quenched by heating to 100°C for 1 min and cooled on ice. A 2% ninhydrin solution was added, and the mixture was vortexed. The reaction was heated to 80°C for 15 min followed by cooling on ice until the solution was 30°C. The absorbance was recorded at 570 nm on a BioTek Syngen microplate reader. Glutamic acid concentrations of 0, 0.1, 0.2, 0.3, 0.4, and 0.5 mM were used as standards for primary amine development with ninhydrin.

#### IC_50_ determinations

IC_50_ values were fitted with a program that uses a non-linear least squares regression of the Gauss-Newton algorithm with optional damping using an ad hoc program.[16,17] All inhibition assays were conducted with a reaction volume of 200 μL, 2 mM **1b** and 8 nM *Hi*DapE unless otherwise stated. Glutamic acid standards of 0 to 0.5 mM were used in every trial. To a 50 mM HEPES, pH 7.5 buffered solution at 30°C was added the desired inhibitor followed by *Hi*DapE and incubated for 10 min. The *N*^6^-methyl-L,L-SDAP substrate **1b** was added and allowed to react for 10 min followed by heating to 100°C for 1 min and cooled on ice to 30°C. A 2% ninhydrin solution (100 μL) was added and the mixture was vortexed. The reaction was heated to 80°C for 15 min followed by cooling on ice until the solution was 30°C. The absorbance of 80 μL was recorded at 570 nm on a BioTek Syngen microplate reader.

## Results and discussion

### *Hi*DapE molecular modeling to assess L,L-SDAP derivatives

An X-ray crystal structure of *Hi*DapE bound by the products of hydrolysis, succinic acid and L,L-DAP, (PDB: 5VO3) revealed a previously unknown closed conformation for the α_2_ dimer.[24] This closed conformation effectively precludes access to the dinuclear Zn(II) active site and defines a negatively charged, crescent-shaped cavity in which the products are bound. We proposed that the presence of substrate induces a conformational change in the enzyme structure that facilitates catalytic activation within the active site. Without the substrate present, the enzyme remains in the open, inactive conformation. In effect, the substrate acts as the “glue” that links the catalytic domain of chain A to the dimerization/cap domain of chain B. Therefore, this products-bound structure[24] was utilized as the structural model for substrate and substrate analog docking experiments.

**Fig 2:**
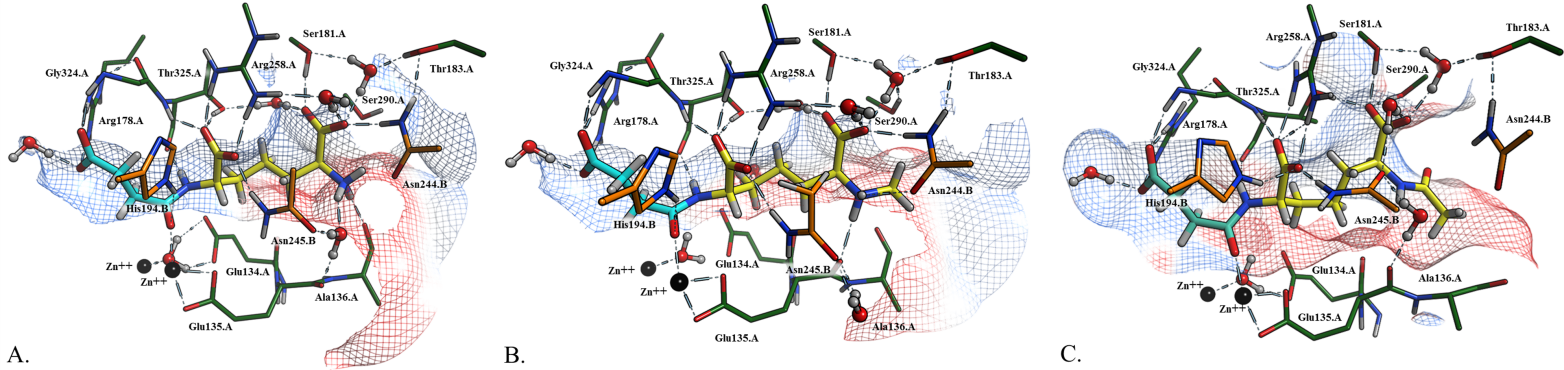
Minimized substrate analogs docked and modeled in the *Hi*DapE active site. The diaminopimelate moiety is depicted in yellow and the succinate in turquoise. A) Native substrate L,L-SDAP, B) *N*^6^-methyl-L,L-SDAP, and C) *N*^6^-acetyl-SDAP. The catalytic domain of Chain A is depicted in green, whereas the dimerization domain of Chain B is shown in orange.

Binding models for L,L-SDAP (**1a**), *N*^6^-methyl-L,L-SDAP (**1b**), and *N*^6^-acetyl-L,L-SDAP (**1c**) were built using the Molecular Operating Environment (MOE) computational suite’s Builder utility followed by minimization in the gas phase using the force field MMFF94X. Following receptor preparation, molecular docking was performed using previously-generated ligand conformation databases to obtain the three substrate-bound enzyme models. Each model was solvated in a simple water box at pH 7.4 and the system's atoms were optimized with a short, localized molecular minimization process. Molecular Dynamics parameters were set to globally minimize the protein, ligand, and solvent atoms with a typical heating and cooling protocol. Simulation results were then minimized once again before the final binding poses were obtained for comparison (Fig 2).

Docking poses for L,L-SDAP (**1a**) and its analogs **1b** and **1c** were assessed as judged by their similarity to product binding interactions.[24] For L,L-SDAP, the amide carbonyl is bound to Zn2 while the amide N-H acts as an H-bond donor to the backbone carbonyl of Thr325:A (Fig 2A). The proximal carboxylate participates in bifurcated H-bonds with the side chains of Arg258:A, Thr325:A, and Asn245:B. The free primary amino group acts as an H-bond donor to the backbone carbonyl of Ala136:A and a water molecule, which in turn participates in H-bond donation to the backbone carbonyl of Glu135:A and to the side chain carbonyl of Asn245:B. The terminal carboxylate of the pimelic acid moiety is H-bonded to the N-H of Asn244:B, the side chain hydroxyl of Ser181:A and Ser290:A, and a water molecule, which in turn H-bonds to the side chain hydroxyls of Thr183:A and Thr325:A.

Interactions between the *N*^6^-methyl-L,L-SDAP analog **1b** with enzyme sidechain residues are identical to those of L,L-SDAP, except that the added methyl group on the primary amine eliminates the H-bonding interaction between the amine and the backbone carbonyl of Ala136:A (Fig 2B). The presence of the *N*^6^-methyl group also leads to perturbations between the ammonium N-H and the corresponding water molecule. On the other hand, the H-bonding interaction between the backbone carbonyl of Glu135:A and the sidechain carbonyl of Asn245:B, by the ammonium N-H(s), are maintained. Interestingly, the *N-*methyl forms a new hydrophobic interaction with the adjacent Ala136:A residue. The model of the *N-*acetyl derivative **1c** is affected both by the lack of the positive charge on the nitrogen as well as the greater steric demand of the acetyl group. The pocket is pushed open as indicated by the surface map, and the His194.B, a key catalytic residue from the remote chain recently implicated in the mechanism,[24] is no longer interacting the amide carbonyl of the substrate, but is interacting with the carboxylate instead. All stabilizing H-bond interactions with the *N*^6^-H groups are lost in the neutral acetamide derivative, and the presence of the acetyl group has forced Asn224.B from its H-bonding interaction with the carboxylate of the substrate analog. Significantly, Glu134 is proposed to act as the general acid/base during the hydrolysis reaction catalyzed by *Hi*DapE[28] and this residue is shifted further away from the active site, likely impeding the enzyme’s ability to hydrolyze the substrate. Furthermore, it should be emphasized that these models were calculated using the closed conformation of DapE[24] rather than the open form,[9] and that poor substrate analogs such as acetamide **1c** may well not even induce the large conformational change that the DapE enzyme undergoes when acting upon its substrate.

### Chemistry

**Fig 3:**
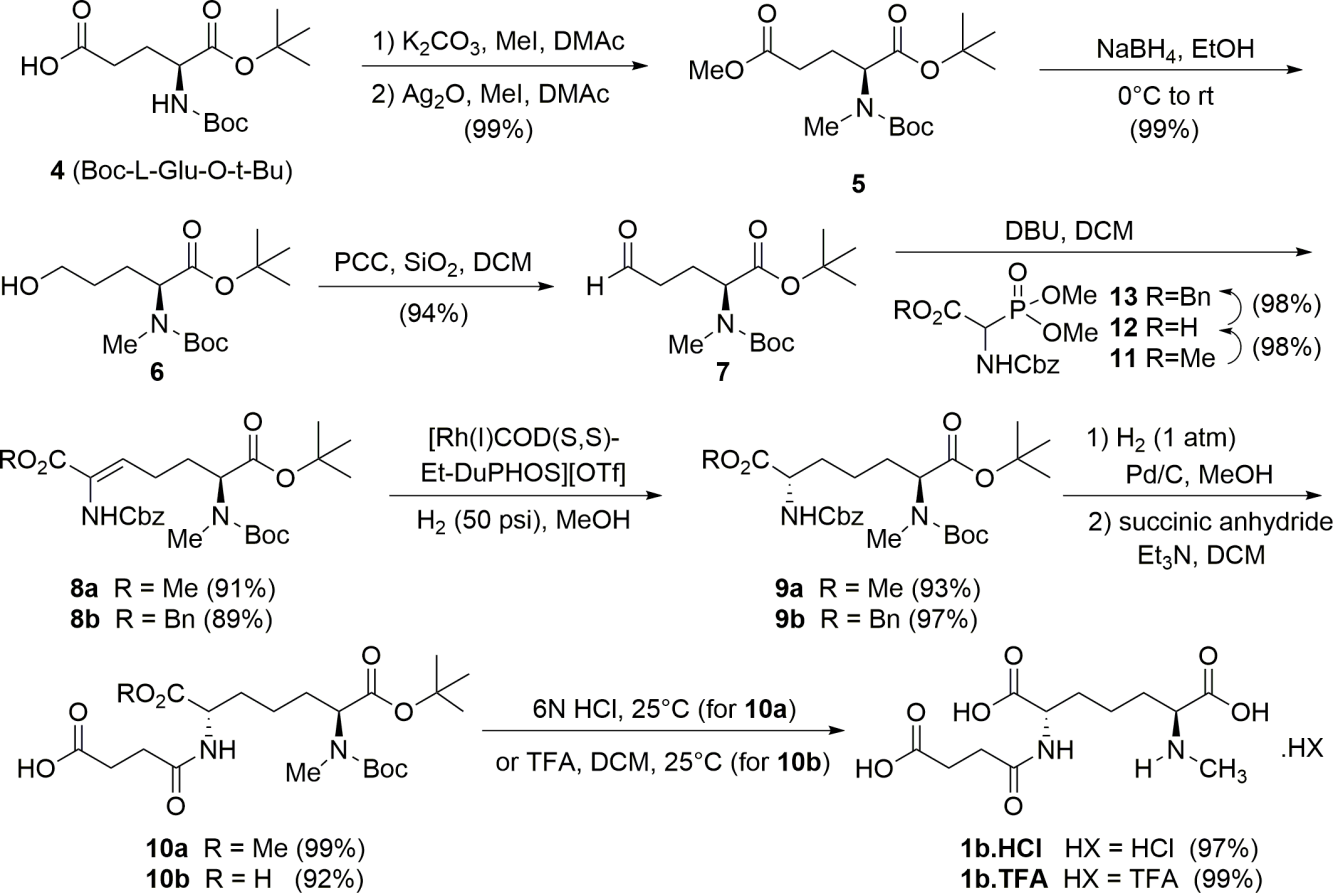
Asymmetric synthesis of *N*^6^-methyl-L,L-SDAP 1b. Synthetic route for preparation of monomethyl substrate analog as the hydrochloride salt (**1b.HCl**) via the methyl ester or the trifluoroacetate salt (**1b.TFA**) via the benzyl ester.

Based on these molecular modeling data, *N*^6^-methyl-L,L-SDAP **1b** is predicted to retain an overall similar binding pose to L,L-SDAP **1a**, making it a possible substrate analog, while *N*^6^-acetyl-L,L-SDAP **1c** suffers significant binding alterations due to loss of the positive charge and the presence of the acetyl group, and N^6^,*N*^6^-dimethyl-L,L-SDAP was also rejected, as mentioned. Therefore, *N*^2^-succinyl-*N*^6^-methyl-L,L-DAP **1b** was prepared enantioselectively, as illustrated in Fig 3, with the key olefination and asymmetric hydrogenation steps modeled after Hruby’s[29] synthesis of diaminopimelic acid, which lacks the *N*^6^-methyl as well as the succinate. In addition, the present route avoids any use of ion exchange chromatography that was used in Hruby’s smaller-scale work. The first step involves the one-pot methylation of the BOC-L-glutamic acid *t*-butyl ester **4** with potassium carbonate and methyl iodide, in the presence of silver oxide, to afford the α-*N-*methylated ester **5**. Reduction of the methyl ester with DIBAL gave mixtures of aldehyde, alcohol, and unreacted methyl ester, so the methyl ester was reduced with sodium borohydride to afford the primary alcohol **6**, which was then oxidized to the aldehyde **7** with PCC or PDC. Horner-Wadsworth-Emmons olefination with Cbz-α-phosphonoglycine ester (Cbz = carboxybenzyl) gave the olefin **8a**, which was enantioselectively hydrogenated in the presence of catalytic amounts of 1,2-bis[(2*S*,5*S*)-2,5-diethylphospholano]benzene(1,5-cyclooctadiene)rhodium(I) trifluoromethanesulfonate affording the L,L-Cbz-protected amino acid **9a** in 93% yield. The olefination followed by the asymmetric hydrogenation and catalyst selection follow the work of Hruby, as noted.[29] Removal of the Cbz protecting group by hydrogenolysis followed by reaction with succinic anhydride afforded the succinamide derivative **10a** in 99% yield, which was subjected to hydrolysis with aqueous HCl to afford *N*^6^-methyl-L,L-SDAP as the hydrochloride salt (**1b.HCl**) in 97% yield, in contrast to using HBr[29] which also cleaved the succinate.

A second route was also developed that was an improvement, based on yield, over the initial methyl ester route by utilizing a benzyl ester. In this synthetic scheme, the Wadsworth-Emmons reaction of the aldehyde **7** with benzyl 2-{[(benzyloxy)carbonyl]amino}-2-(dimethoxyphosphoryl)acetate (R = Bn) afforded olefin **8b**, which was enantioselectively hydrogenated in the presence of catalytic 1,2-bis[(2*S*,5*S*)-2,5-diethylphospholano]benzene(1,5-cyclooctadiene)rhodium(I) trifluoromethanesulfonate to afford the L,L-Cbz-protected amino acid benzyl ester **9b** in 97% yield. Hydrogenolytic removal of both the benzyl and Cbz groups followed by reaction with succinic anhydride gave the succinate amide (92–97% for **10b)**. Removal of both *tert*-butyl ester and Boc groups with trifluoroacetic acid in methylene chloride gave the trifluoroacetate salt of **1b** in nearly quantitative yield. Overall, the benzyl ester route proved to be more amenable for large scale reactions, although the intrinsic substrate diastereoselectivity for asymmetric reduction of the methyl ester yielded material of 97.4% e.e. for methyl ester **9a**, compared to 92.0% e.e. for the benzyl ester **9b**. Nevertheless, crystallization at a later stage ultimately afforded the final product **1b** in >99% e.e. from the benzyl ester, whereas the methyl ester was often accompanied by some desuccinylation. Furthermore, the benzyl ester route provides the TFA salt which was not observed to be hygroscopic, in contrast to the hydrochloride salt from the methyl ester route which is very hygroscopic and converts to a sticky and unweighable solid over time in storage.

Having synthesized *N*^6^-methyl-L,L-SDAP **1b**, the ability of *Hi*DapE to hydrolyze this proposed substrate analog was examined by monitoring amide bond hydrolysis at 225 nm in 50 mM HEPES buffer at pH 7.5. As predicted *via* molecular modeling, *Hi*DapE was capable of hydrolyzing **1b**, and a k_cat_/K_m_ of 3.87x 10^4^ M^-1^S^-1^ was measured, compared to the endogenous L,L-SDAP substrate with a k_cat_/K_m_ of 1.92 x 10^5^ M^-1^ S^-1^. *N*^6^-Acetyl-L,L-SDAP (**1c**) was also prepared by direct acylation of L,L-SDAP. As expected based on modeling, **1c** was not hydrolyzed by *Hi*DapE, likely due to the need for a cationic species at the *N*^6^ position as well as unfavorable steric interactions.

### Primary amine detection with ninhydrin

Since **1b** can function as a substrate, we set out to design a robust and operationally straightforward enzymatic assay, utilizing ninhydrin, that would be amenable to inhibition studies for drug discovery. It should be noted that ninhydrin has been employed for enzymatic product detection in a coupled assay to identify inhibitors of glycopeptide resistance among a library of over 250,000 compounds.[30] While ninhydrin reacts with both primary and secondary amines, only primary amines that bear an alpha-hydrogen can form the Schiff base known as Ruhemann's purple, which absorbs with λ_max_ values of 570 and 450 nm. In contrast, ninhydrin reacts with secondary amines such as proline to form an iminium salt that is yellow-orange in color with λ _max_ values of 440 and 405 nm.[31] Initial control reactions were performed with ninhydrin in the absence of enzyme by monitoring absorptions between 300 and 800 nm to determine the optimal time of heating and color development. Glutamic acid was utilized as a model of a primary amine while *N-*methylglycine (sarcosine) was selected as a model for acyclic secondary amine substrates, such as **1b**. Heating of these model primary and secondary amines with ninhydrin at 100°C for 15 min provided very similar absorption spectra with identical λ_max_ values of 404 and 570 nm, due the products of ninhydrin with glutamic acid and sarcosine, respectively (S2 Fig). However, some secondary amines are known to react with ninhydrin in the presence of polar aprotic solvents such as DMSO, the solvent in which the ninhydrin reagent is provided.[32]

As absorption from the secondary amine of **1b** could potentially interfere with the accurate determination of the primary amine produced from enzymatic hydrolysis, the slower rate of reaction of the secondary amine with ninhydrin relative to the primary amine was examined. Reducing the time of incubation of the model substrates glutamic acid and sarcosine with ninhydrin at 100°C to only 2 min (S2 Fig) provided significantly greater absorption at 570 nm, indicating preference for the primary amine over the secondary amine—a difference that diminished upon continued heating of the samples to 15 min. These data suggest that a shorter incubation time of ca. 2 min might enable a clear distinction between the primary and secondary amines of the **1b** product of hydrolysis, compound **3b**. However, at 2 min even the primary amine was not completely reacted resulting in increased error. Another practical challenge of a ~2 min incubation time prompted the examination of lower incubation temperatures to enable full reaction of the primary amine with ninhydrin while minimizing the reaction of the secondary amine. The first directed approach was to monitor product absorption development over 15 min at varying temperatures in 20°C increments. Time course plots of the reaction with glutamic acid or sarcosine (1.0 mM) and ninhydrin (S3 Fig) show positive reactions with the primary amine at 80°C and 60°C, whereas little or no detectable reaction is observed with the secondary amine at these temperatures. Absorbance due to primary and secondary amines at these lower temperatures reaches a maximum at ~15 min. These data demonstrate that heating the amine reaction mixture at 80°C for 15 min is optimal with an absorbance of ≤1 AU for the primary amine product that is easily detectable, with no significant absorption due to ninhydrin reaction with the secondary amine.

### Quenching of *Hi*DapE hydrolytic activity

To develop a functional spectrophotometric assay for DapE enzymes, it is essential to confirm that the enzyme activity is effectively quenched before ninhydrin is added. As ninhydrin is a commercially available 2% solution in DMSO, both the addition of DMSO and the elevated reaction quench temperature were examined independently to ensure that the enzymatic reaction was stopped. DMSO is known to decrease or halt enzymatic activity, due to denaturation, in the presence of as little as 10% by volume.[33] Therefore, control reactions of standard enzyme activity were carried out in triplicate as follows: to a buffered *Hi*DapE solution at 30°C was added **1b** as the TFA (trifluoroacetate) salt. The reaction was allowed to proceed for ~10 min after which the 2% ninhydrin reagent in DMSO was added and subsequently heated to 80°C for 15 min. The final assay volume was 300 μL after the addition of 100 μL of ninhydrin reagent giving a final concentration of 33 vol% of DMSO. The ninhydrin reaction was quenched by placing the mixture on ice for ~2 min after which the absorbance was determined at 570 nm. This control reaction was set as 100% enzymatic activity of *Hi*DapE.

*Hi*DapE was incubated with DMSO prior to the addition of **1b** under various times to examine the effects of DMSO on the observed hydrolytic activity. The enzyme was allowed to incubate for various times between 0 and 10 min after which **1b** was added and the reaction allowed to proceed for ~10 min. The enzymatic reaction was quenched by addition of 2% ninhydrin solution and was subsequently heated to 80°C for 15 min. The ninhydrin reaction was quenched by placing on ice for ~2 min after which the absorbance at 570 nm was determined. Comparison of standardized *Hi*DapE activity to those obtained in the presence of DMSO indicate a ca. 50% decrease. Interestingly, the observed enzymatic activity is not abolished even after 10 min. These data demonstrate that the addition of a ninhydrin/DMSO solution does not fully quench the catalytic activity of *Hi*DapE.

#### Pre-heat incubation studies

The effect of heating on the enzymatic activity of *Hi*DapE was examined by pre-heating *Hi*DapE at 80°C and 100°C in increments from 0 to 10 min, followed by cooling to room temperature. The enzymatic activity of these samples was determined at 25°C by the addition of **1b**. Incubating *Hi*DapE at 80°C for various times between 0 and 5 min before the addition of **1b** reveals a dramatic decrease in enzymatic activity within 2 min with no detectable enzymatic activity observed after 2 min. Incubating *Hi*DapE at 100°C completely eliminated its catalytic ability within 1 min (S5 Fig). However, heating to 100°C also causes both primary and secondary amines to react at an appreciable rate with ninhydrin. Combination of these data with model and standardized *Hi*DapE ninhydrin reaction data indicate that heating *Hi*DapE to ~80°C quenches the enzyme activity within 2 min, which also allows only the primary amine from the cleaved substrate to react with the ninhydrin. Therefore, to minimize the associated error in any spectrophotometric assay for *Hi*DapE, the enzymatic activity can be quenched by heating at 100°C before the addition of the ninhydrin/DMSO reagent followed by subsequent development at 80 ^o^C.

#### Circular dichroism denaturation studies

**Fig 4:**
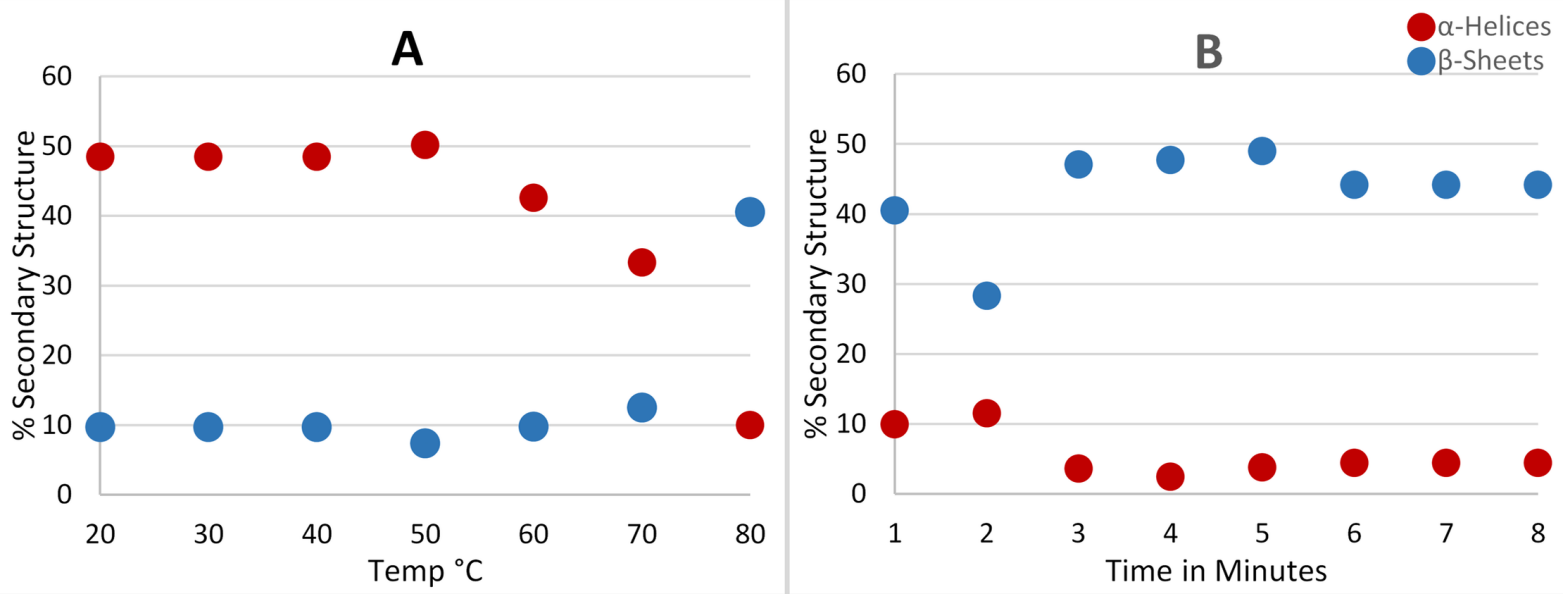
Circular dichroism thermal denaturation study of *Hi*DapE. The α-helical structures are represented in red and β-sheets are represented in blue with (A) percent secondary structure observed over the course of heating from 20–80 *°*C, and (B) percent secondary structure remaining with continued heating at 80 *°*C.

Denaturation of *Hi*DapE upon heating was confirmed by Circular Dichroism (CD) spectroscopy recorded as a function of temperature (Fig 4). Based on activity data, a ~5% loss of α-helical structure was used as an indication that *Hi*DapE was unfolding while ≤10% total remaining α-helical structure indicated complete denaturation of *Hi*DapE. At ~60°C, *Hi*DapE begins to denature as evidenced by a ~15% reduction in α-helical secondary structure. Upon heating to 80°C, the observed α-helical structure decreased to ~10%. These data confirm that complete denaturation of *Hi*DapE occurs after ~2 min of heating at 80°C.

### Enzyme kinetics for *N*^6^-methyl-L,L-SDAP 1b

**Fig 5:**
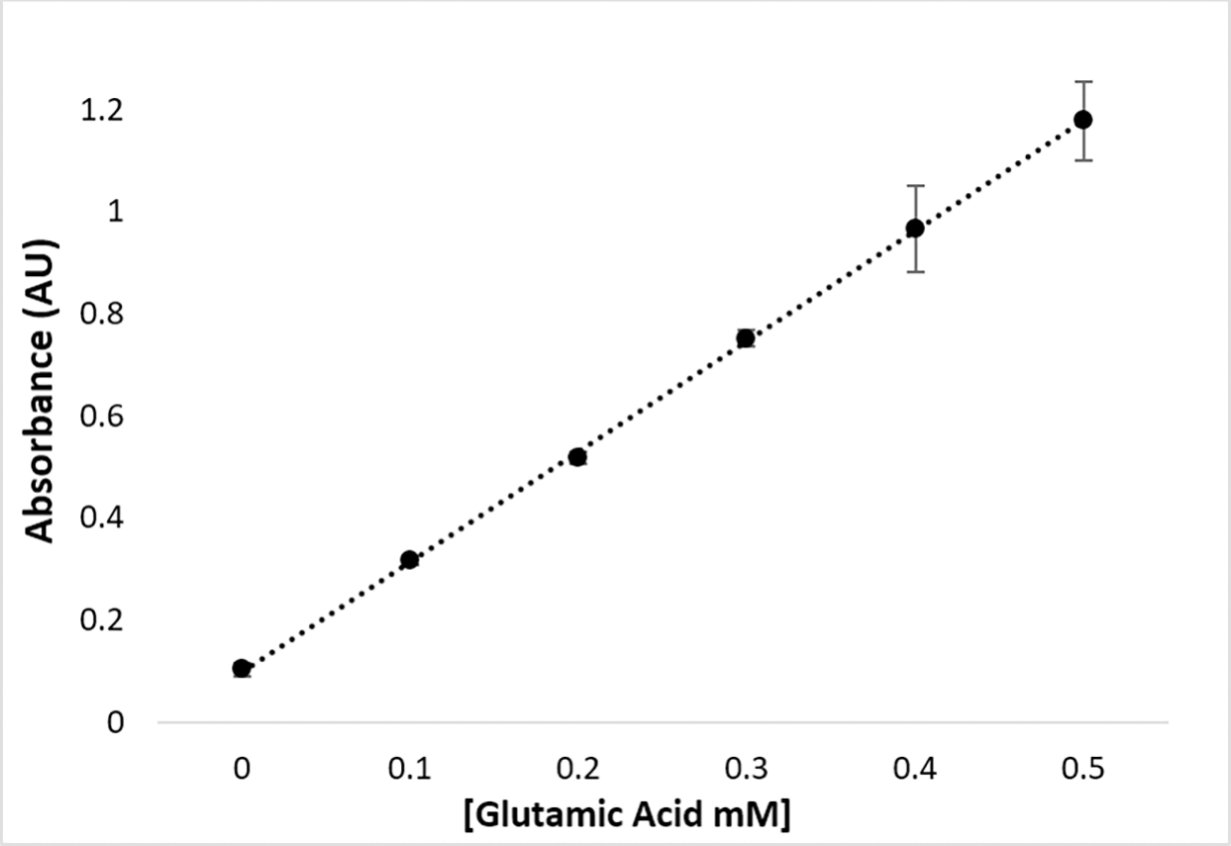
Glutamic acid control standard curve for the development of ninhydrin and primary amine in 50 mM HEPES buffer at pH 7.5.

**Fig 6:**
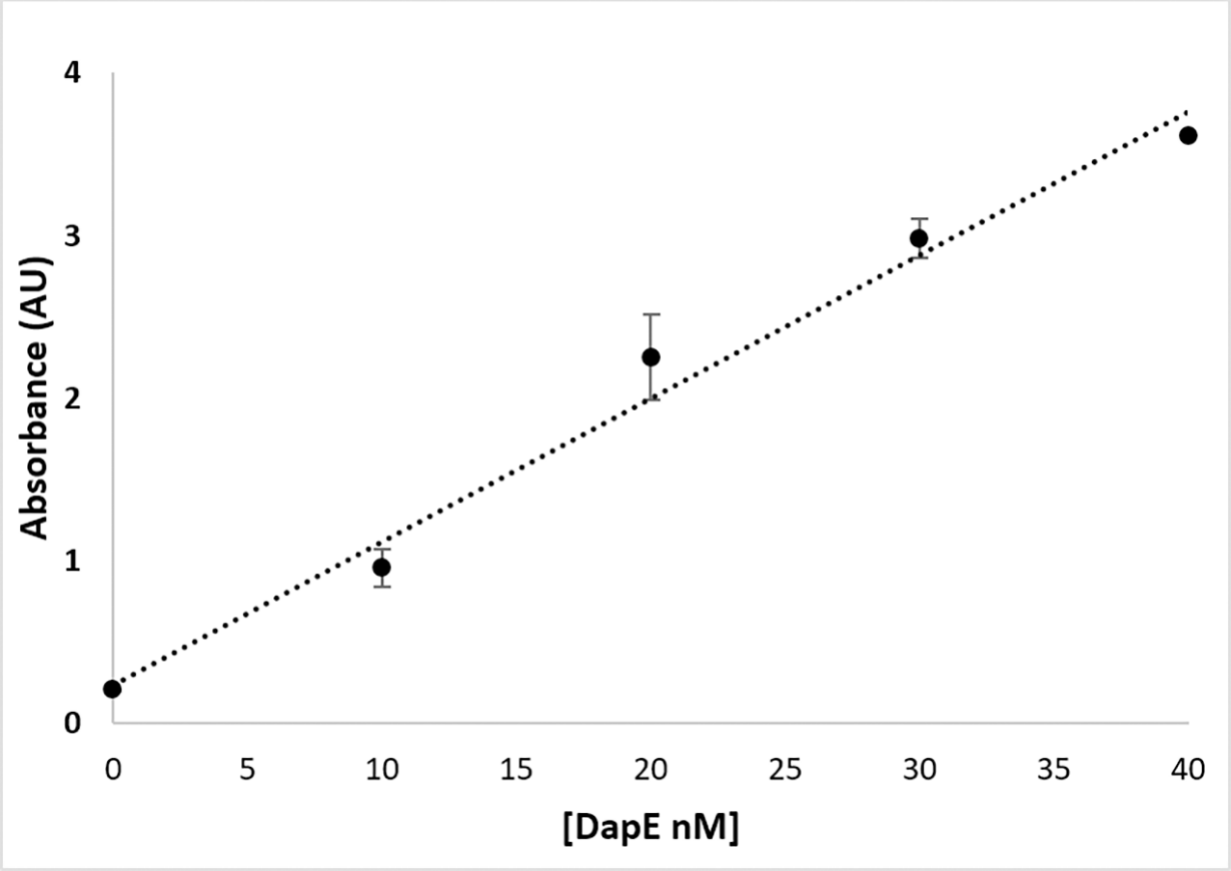
Enzyme saturation curve of *Hi*DapE using 2 mM of *N*^6^-methyl-L,L-SDAP as the substrate (50 mM HEPES buffer at pH 7.5). Optimal enzyme concentration selected for absorbance of primary amine product at or around 1 AU (absorbance unit).

The optimized experimental assay conditions enable the detection of products as well as providing proper conditions for quenching *Hi*DapE hydrolytic activity. The next step in developing a spectrophotometric assay for *Hi*DapE that can be used to screen potential inhibitors via high-throughput screening is the normalization of the reactant concentrations. Utilizing glutamic acid as a model of a primary amine, a concentration of 0.4 mM of glutamate reacted with ninhydrin produced an absorbance of ~1 AU at 570 nm (Fig 5). Based on these data, *Hi*DapE concentrations were examined as a function of product formation using **1b** as the substrate in 50 mM HEPES buffer, pH 7.5 at 30°C. A concentration of ~2 mM of **1b** with a *Hi*DapE concentration of 8 nM produces ~80 nmol of product in 10 min. The product can be detected colorimetrically by the addition of 2% ninhydrin (100 μL) followed by heating at 80°C for 15 min and cooling to 30°C resulting in an absorbance of ~1 AU at 570 nm (Fig 6). Importantly, the rate of the enzymatic reaction at 10 min lies within the linear portion of the curve. These data indicate that the use of **1b** as the substrate followed by colorimetric development of the primary amine product with ninhydrin provides a robust and reproducible assay for DapE enzymes.

### *Hi*DapE ninhydrin-based enzymatic assay and IC_50_ determination

To test **1b** as a substrate for screening potential inhibitors of *Hi*DapE, the potency of several previously identified inhibitors was examined. Specifically, captopril was found to have an IC_50_ value of 3.4 ±0.2 μM using the ninhydrin based assay described herein (S6 Fig), which is almost identical to that reported using L,L-SDAP as the substrate and measuring cleavage of the substrate at 225 nm (IC_50_ = 3.3 μM).[10] Interestingly, captopril displayed modest antibiotic activity against *Escherichia coli* and *Salmonella enterica*[34] confirming our own observation of antibiotic activity.[10] However, Uda found that captopril does not inhibit the Mn(II) form of the enzyme,[35] and that, surprisingly, the antibiotic activity of captopril was independent of DapE inhibition in bacteria.[34] Ultimately, it will be imperative to screen different metalloisozymes of DapE enzymes, in particular the Mn(II) isoform in addition to the Zn(II) bound enzyme, as captopril was found to be a moderately potent inhibitor of the Zn(II) enzyme but not of the Mn(II) enzyme. Moreover, mono- and di-Zn(II) enzymes were shown to exhibit different rates of hydrolysis of L,L-SDAP and their crystal structures suggest different approaches will be required to rationally design inhibitors specific for each form.[9] The new ninhydrin-coupled DapE assay will simplify such experiments and assist in the screening and design of inhibitors based on captopril. Likewise, the IC_50_ values for 3-mercaptobenzoic acid (IC_50_ = 21.8 ± 2.2 μM), phenylboronic acid (IC_50_ = 316 ± 23.6 μM), and thiophene boronic acid (IC_50_ = 111 ± 16 μM) were found to be in good agreement with the literature values of 35, 107, and 92 μM, respectively (S7–S9 Figs).[10] These data confirm that the ninhydrin based *Hi*DapE assay based on **1b** as the substrate provides comparable results to the standard L,L-SDAP assay that directly monitors the amide bond cleavage at 225 nm.

#### Ninhydrin-based DapE assay tolerance for amines

Amines are critically important functional groups in drugs and in compound screening libraries. We were therefore very concerned that primary amines might appear as false positives in an inhibitor library screen due to their reactivity with ninhydrin, so we screened benzylamine, cyclohexylamine, and aniline as potential inhibitors in the assay. At a concentration of 200 μM, none of these amines inhibit or give the appearance of inhibiting DapE using this assay. A blank control of these primary amines from 100 μM up to 500 μM demonstrates that these primary amines are not detected at 570 nm, consistent with the fact that their reaction with ninhydrin does not produce the Ruhemann’s purple complex that absorbs at 570 nm, and instead produces an iminium salt complex which is not detected at this wavelength. In addition, we screened glutamic acid as a potential inhibitor of DapE at 200 μM, and glutamic acid does not appear to inhibit the DapE enzyme at this concentration, even though it is an alpha-amino acid that can produce Ruhemann’s purple, due to subtraction of the blank.

## Conclusion

In summary, *N*^6^-methyl-L,L-SDAP **1b** was predicted to be a potential substrate for *Hi*DapE whereas *N*^6^-acetyl-L,L-SDAP **1c** was shown to suffer significant binding alterations due to the larger acetyl group and loss of charge on the nitrogen of the amide. Two synthetic routes to **1b** were developed allowing the synthesis of **1b** in very high yields on a multigram scale. Kinetic studies proved that **1b** can be hydrolyzed by *Hi*DapE at comparable rates to L,L-SDAP, establishing **1b** as a substrate for DapE enzymes. Another potential substrate analog, *N*^6^-acetyl-L,L-SDAP (**1c**), was also synthesized and tested as a substrate for *Hi*DapE, but as expected, no activity was detected. Since the amine-containing product of the hydrolysis of **1b** contains only one primary amine, conditions that selectively modify the primary amine *vs*. the secondary amine with ninhydrin were developed, resulting in a new spectrophotometric assay for DapE enzymes that is inexpensive, robust and reproducible. The newly discovered DapE substrate, **1b**, coupled with ninhydrin development, is superior to the standard UV-based (225 nm) assay which uses L,L-SDAP as the substrate, as it is alleviates interference from potential inhibitor absorption at 225 nm, a common absorption region for aryl containing compounds. The feasibility of the new **1b**-ninhydrin-coupled DapE assay was established by accurately determining IC_50_ values of known *Hi*DapE inhibitors. In summary, this new ninhydrin-based assay provides a method for the discovery of lead compounds for DapE enzymes and for driving medicinal chemistry structure-activity relationships (SAR) and thus provide a critical tool for the discovery of a new class of antibiotics that target DapE enzymes.

## Supporting information

S1 Fig Minimized *N*^6^,*N*^6^-dimethyl-L,L-SDAP substrate analog docked and modeled in the *Hi*DapE active site. The diaminopimelate moiety is depicted in yellow and the succinate in turquoise. The catalytic domain of Chain A is depicted in green, whereas the dimerization domain of Chain B is shown in orange. *N*^6^,*N*^6^-Dimethyl-L,L-SDAP binds quite distinctly compared to L,L-SDAP due to the presence of the additional methyl groups. Loss of the interfacial domain interaction between the backbone carbonyl of Glu135:A and the side chain carbonyl of Asn245:B by the ammonium N-H species, due to interference of the additional methyl groups, appear to be the key differences. The additional bulk of the two methyl groups also leads to the migration of the N-H bond from the ammonium species from Glu135:A to the backbone carbonyl of Glu134:A. Significantly, Glu134 is proposed to act as the general acid/base during the hydrolysis reaction catalyzed by *Hi*DapE and this residue is shifted further away from the active site, likely impeding the enzyme’s ability to hydrolyze the substrate.
(TIF)

S2 Fig Sarcosine and glutamic acid development with ninhydrin UV/Vis spectra. UV/Vis spectra of sarcosine (secondary amine) and glutamic acid (primary amine) with ninhydrin after heating at A) 100°C for 2 min, and B) 80°C for 2 min.
(TIF)

S3 Fig Time course plots for the development of primary amine and secondary amine at 60°C vs 80°C vs 100°C. Time course plots of the development of primary amine or secondary amine with ninhydrin **(A)** at 100°C (**B**) vs 80°C and (**C**) vs 60°C. Glutamic acid was used as model primary amine, and sarcosine was used as a model secondary amine.
(TIF)

S4 Fig Circular dichroism UV spectra of denaturation of *Hi*DapE. Circular Dichroism UV/Vis spectra of thermal denaturation of *Hi*DapE observing the α-helical secondary structure.
(TIF)

S5 Fig Pre-heat incubation of *Hi*DapE at 80°C vs 100°C. Pre-heat incubation reaction of *Hi*DapE at 80°C vs 100°C compared to 100% enzymatic activity and 0% enzymatic activity.
(TIF)

S6 Fig Inhibition of DapE by captopril. (TIF)

S7 Fig Inhibition of DapE by 3-mercaptobenzoic acid. (TIF)

S8 Fig Inhibition of DapE by phenylboronic acid. (TIF)

S9 Fig Inhibition of DapE by 2-thiophene boronic acid. (TIF)

## References

[1] World Health Organization. Antibacterial agents in clinical development: an analysis of the antibacterial clinical development pipeline, including tuberculosis. 2017. http://www.who.int/medicines/areas/rational_use/antibacterial_agents_clinical_development/en/

[2] Klevens RM, Morrison MA, Nadle J, Petit S, Gershrnan K, Ray S, et al Invasive methicillin-resistant *staphylococcus aureus* infections in the United States. JAMA, J Am Med Assoc. 2007; 298: 1763–1771. 10.1001/jama.298.15.1763 17940231

[3] Howe RA, Bowker KE, Walsh TR, Feest TG, MacGowan AP. Vancomycin-resistant *Staphylococcus aureus*. Lancet. 1998; 351: 602 doi: 10.1016/S0140-6736(05)78597-4 9492814 10.1016/S0140-6736(05)78597-4

[4] Gillner DM, Becker DP, Holz RC. Lysine biosynthesis in bacteria: a metallodesuccinylase as a potential antimicrobial target. JBIC, J Biol Inorg Chem. 2013; 18: 155–163. doi: 10.1007/s00775-012-0965-1 23223968 10.1007/s00775-012-0965-1 PMC3862034

[5] Scapin G, Blanchard JS. Enzymology of bacterial lysine biosynthesis. Adv Enzymol Relat Areas Mol Biol. 1998; 72: 279–324. 9559056 10.1002/9780470123188.ch8

[6] Karita M, Etterbeek ML, Forsyth MH, Tummuru MKR, Blaser MJ. Characterization of *Helicobacter pylori* dapE and construction of a conditionally lethal dapE mutant. Infect Immun. 1997; 65: 4158–4164. 9317022 10.1128/iai.65.10.4158-4164.1997 PMC175598

[7] Pavelka MS Jr., Jacobs WR Jr. Biosynthesis of diaminopimelate, the precursor of lysine and a component of peptidoglycan, is an essential function of *Mycobacterium smegmatis*. J Bacteriol. 1996; 178: 6496–6507. 8932306 10.1128/jb.178.22.6496-6507.1996 PMC178536

[8] Cosper NJ, Bienvenue DL, Shokes JE, Gilner DM, Tsukamoto T, Scott RA, et al The dapE-encoded N-Succinyl-L,L-Diaminopimelic Acid Desuccinylase from *Haemophilus influenzae* is a Dinuclear Metallohydrolase. J Am Chem Soc. 2003; 125: 14654–14655. doi: 10.1021/ja036650v 14640610 10.1021/ja036650v

[9] Nocek BP, Gillner DM, Fan Y, Holz RC, Joachimiak A. Structural Basis for Catalysis by the Mono- and Dimetalated Forms of the dapE-Encoded *N*-succinyl-L,L-Diaminopimelic Acid Desuccinylase. J Mol Biol. 2010; 397: 617–626. doi: 10.1016/j.jmb.2010.01.062 20138056 10.1016/j.jmb.2010.01.062 PMC2885003

[10] Gillner D, Armoush N, Holz RC, Becker DP. Inhibitors of bacterial *N*-succinyl-L,L-diaminopimelic acid desuccinylase (DapE) and demonstration of in vitro antimicrobial activity. Bioorg Med Chem Lett. 2009; 19: 6350–6352. doi: 10.1016/j.bmcl.2009.09.077 19822427 10.1016/j.bmcl.2009.09.077

[11] Starus A, Nocek B, Bennett B, Larrabee JA, Shaw DL, Sae-Lee W, et al Inhibition of the dapE-Encoded *N*-Succinyl-L,L-diaminopimelic Acid Desuccinylase from *Neisseria meningitidis* by *L*-Captopril. Biochemistry. 2015; 54: 4834–4844. doi: 10.1021/acs.biochem.5b00475 26186504 10.1021/acs.biochem.5b00475 PMC4671288

[12] Mandal RS, Das S. In silico approach towards identification of potential inhibitors of *Helicobacter pylori* DapE. J Biomol Struct Dyn. 2015; 33: 1460–1473. doi: 10.1080/07391102.2014.954272 25204745 10.1080/07391102.2014.954272

[13] Lin Y, Myhrman R, Schrag ML, Gelb MH. Bacterial *N*-succinyl-L-diaminopimelic acid desuccinylase. Purification, partial characterization and substrate specificity. J Biol Chem. 1988; 263: 1622–1627. 3276674

[14] Kindler SH, Gilvarg C. N-Succinyl-L-α,ε-diaminopimelic acid deacylase. J Biol Chem. 1960; 235: 3532–3535. 13756049

[15] Bienvenue DL, Gilner DM, Davis RS, Bennett B, Holz RC. Substrate specificity, metal binding properties, and spectroscopic characterization of the DapE-encoded *N*-succinyl-L,L-diaminopimelic acid desuccinylase from *Haemophilus influenzae*. Biochemistry. 2003; 42: 10756–10763. doi: 10.1021/bi034845+ 12962500 10.1021/bi034845+

[16] Fraser RDB, Susuki E. Physical Principles and Techniques of Protein Chemistry N.Y.: Academic Press; 1973.

[17] Ziegler AJ, Florian J, Ballicora MA, Herlinger AW. Alkaline phosphatase inhibition by vanadyl-β-diketone complexes: electron density effects. Journal of Enzyme Inhibition and Medicinal Chemistry. 2009; 24: 22–28. doi: 10.1080/14756360701841426 18615288 10.1080/14756360701841426

[18] Glinka T, Lomovskaya O, Bostian K, Wallace DM. Preparation of peptide polybasic bacterial efflux pump inhibitors for enhancing levofloxacin potency in treating bacterial infections. PCT Int Appl. 2008; 2008-US62785; 2007–60917616: 198.

[19] Blatt LM, Pan L, Seiwert S, Andrews SW, Martin P, Schumacher A, et al Preparation of macrocyclic acylsulfonamides, *N*-hydroxy-, *N*-alkoxy- and *N*-aryloxyamides as inhibitors of HCV replication. PCT Int Appl. 2008; 2008-US62552; 2007–60915896: 315.

[20] Jørgensen KB, Gautun OR. Efficient stereoselective preparation of protected isodityrosines. Tetrahedron. 1999; 55: 10527–10536.

[21] Horenstein BA, Nakanishi K. Synthesis of unprotected (±)-tunichrome An-1, a tunicate blood pigment. J Am Chem Soc. 1989; 111: 6242–6246.

[22] Okano K, Tokuyama H, Fukuyama T. Total synthesis of (+)-yatakemycin. J Am Chem Soc. 2006; 128: 7136–7137. doi: 10.1021/ja0619455 16734447 10.1021/ja0619455

[23] Molecular Operating Environment (MOE), 2013.08, Chemical Computing Group Inc., 1010 Sherbooke St. West, Suite #910, Montreal, QC, Canada, H3A 2R7, 2016.

[24] Nocek B, Reidl C, Starus A, Heath T, Bienvenue D, Osipiuk J, et al Structural Evidence for a Major Conformational Change Triggered by Substrate Binding in DapE Enzymes: Impact on the Catalytic Mechanism. Biochemistry (N.Y.). 2018; 57: 574. 10.1021/acs.biochem.7b01151 PMC6886521 29272107

[25] Labute P. The generalized Born/volume integral implicit solvent model: Estimation of the free energy of hydration using London dispersion instead of atomic surface area. Journal of Computational Chemistry. 2008; 29: 1693–1698. doi: 10.1002/jcc.20933 18307169 10.1002/jcc.20933

[26] Shoichet BK. Virtual screening of chemical libraries. Nature. 2004; 432: 862–865. doi: 10.1038/nature03197 15602552 10.1038/nature03197 PMC1360234

[27] Gill SC, Von Hippel PH. Calculation of protein extinction coefficients from amino acid sequence data. Anal Biochem. 1989; 182: 319–326. 2610349 10.1016/0003-2697(89)90602-7

[28] McGregor WC, Swierczek SI, Bennett B, Holz RC. Characterization of the catalytically active Mn(II)-loaded argE-encoded *N*-acetyl-L-ornithine deacetylase from *Escherichia coli*. JBIC, J Biol Inorg Chem. 2007; 12: 603–613. doi: 10.1007/s00775-007-0211-4 17333302 10.1007/s00775-007-0211-4

[29] Wang W, Xiong C, Yang J, Hruby VJ. An efficient synthesis of (2*S*, 6*S*)-and *meso*-diaminopimelic acids via asymmetric hydrogenation. Synthesis. 2002; 2002: 0094–0098.

[30] Pratt SD, Xuei X, Mackinnon AC, Nilius AM, Hensey-Rudloff DM, Zhong P, et al Development of a coupled VanA/VanX assay: screening for inhibitors of glycopeptide resistance. Journal of Biomolecular Screening. 1997; 2: 241–247.

[31] Friedman M. Applications of the Ninhydrin Reaction for Analysis of Amino Acids, Peptides, and Proteins to Agricultural and Biomedical Sciences. J Agric Food Chem. 2004; 52: 385–406. doi: 10.1021/jf030490p 14759124 10.1021/jf030490p

[32] Friedman M, Krull LH. *N*- and *C*-alkylation of peptides and proteins in dimethyl sulfoxide. Biochim Biophys Acta, Protein Struct. 1970; 207: 361–3. 10.1016/0005-2795(70)90028-0 5450135

[33] Yin B, Chen Y, Lin S, Hsu W. Production of *D*-amino acid precursors with permeabilized recombinant *Escherichia coli* with *D*-hydantoinase activity. Process Biochem (Oxford). 2000; 35: 915–921.

[34] Uda NR, Creus M. Selectivity of inhibition of *N*-succinyl-L,L-diaminopimelic acid desuccinylase in bacteria: the product of DapE-gene is not the target of L-captopril antimicrobial activity. Bioinorg Chem Appl. 2011: 306465, 6. 10.1155/2011/306465 PMC3092495 21577314

[35] Uda NR, Upert G, Angelici G, Nicolet S, Schmidt T, Schwede T, et al Zinc-selective inhibition of the promiscuous bacterial amide-hydrolase DapE: implications of metal heterogeneity for evolution and antibiotic drug design. Metallomics. 2014;6: 88–95. doi: 10.1039/c3mt00125c 24057071 10.1039/c3mt00125c

