# Supplementary figures and images for: Practical spectrophotometric assay for the dapE-encoded N-succinyl-L,L-diaminopimelic acid desuccinylase, a potential antibiotic target
Source: PLoS One. 2018 Apr 26;13(4):e0196010. doi: 10.1371/journal.pone.0196010 (PMC5919655; doi:10.1371/journal.pone.0196010)

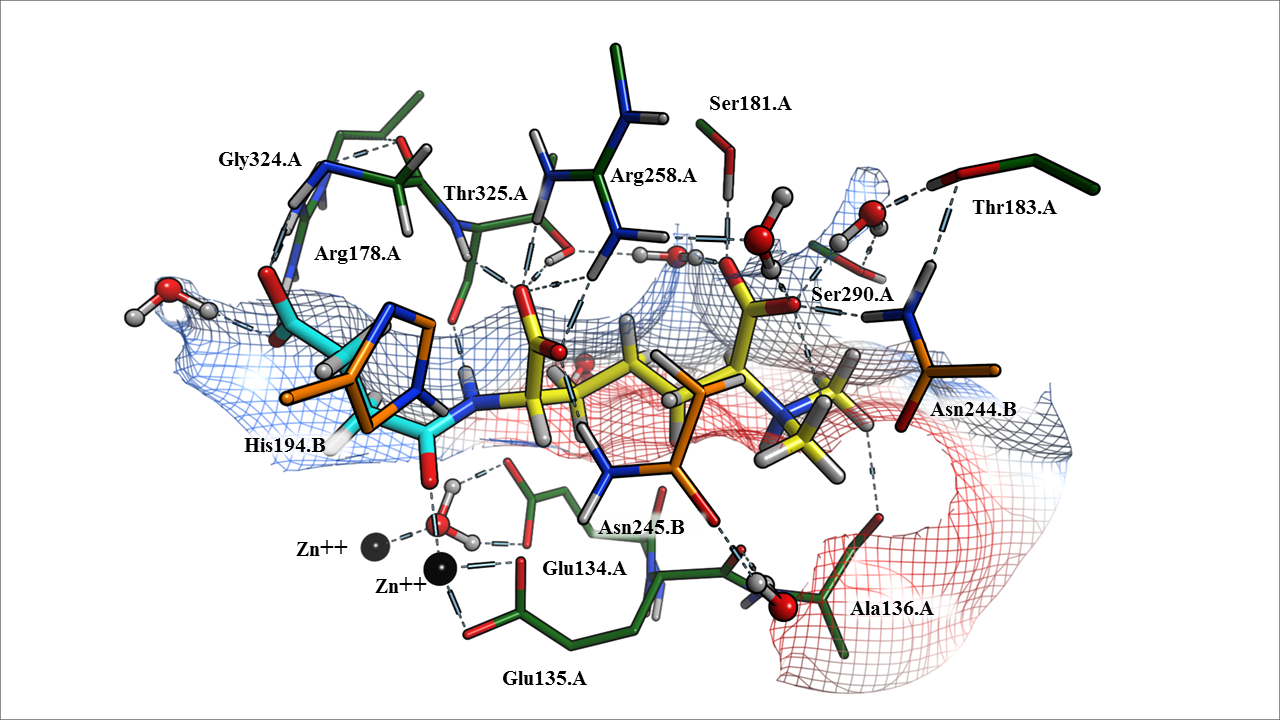

Supplement: S1 Fig — The diaminopimelate moiety is depicted in yellow and the succinate in turquoise. The catalytic domain of Chain A is depicted in green, whereas the dimerization domain of Chain B is shown in orange. N6,N6-Dimethyl-L,L-SDAP binds quite distinctly compared to L,L-SDAP due to the presence of the additional methyl groups. Loss of the interfacial domain interaction between the backbone carbonyl of Glu135:A and the side chain carbonyl of Asn245:B by the ammonium N-H species, due to interference of the additional methyl groups, appear to be the key differences. The additional bulk of the two methyl groups also leads to the migration of the N-H bond from the ammonium species from Glu135:A to the backbone carbonyl of Glu134:A. Significantly, Glu134 is proposed to act as the general acid/base during the hydrolysis reaction catalyzed by HiDapE and this residue is shifted further away from the active site, likely impeding the enzyme’s ability to hydrolyze the substrate. (TIF) [file pone.0196010.s001.tif]

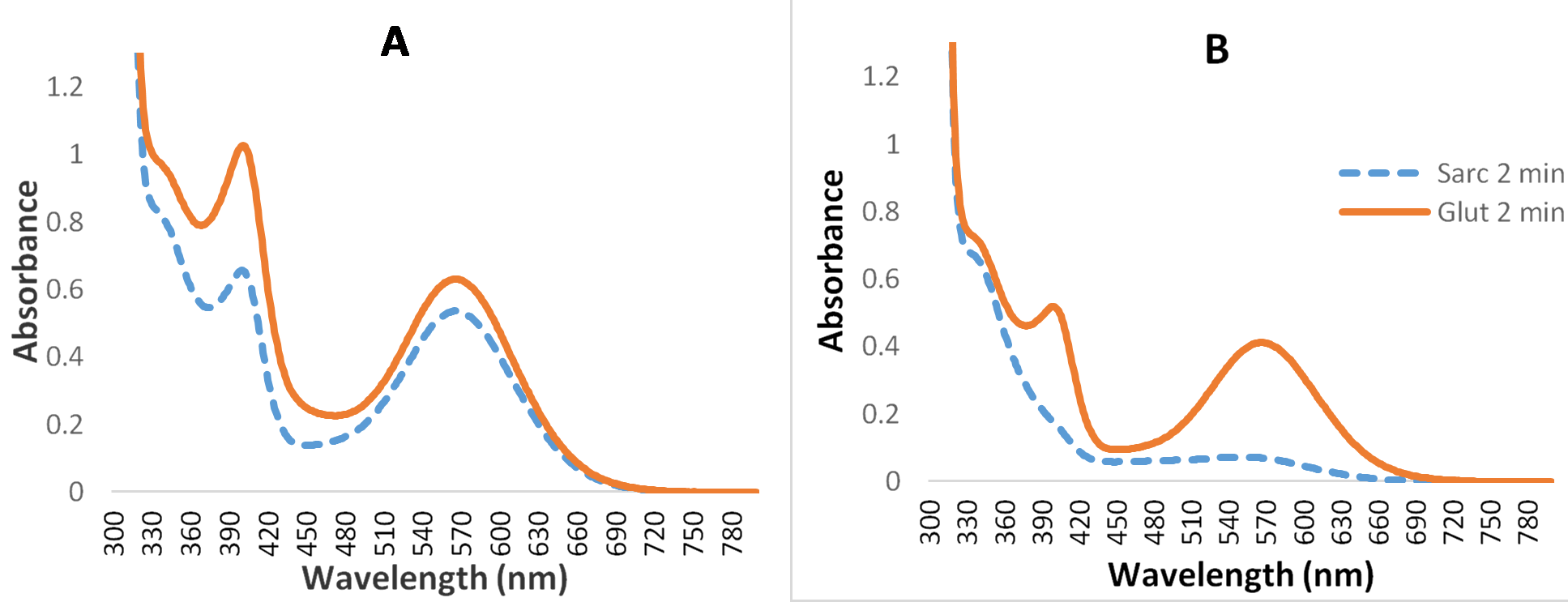

Supplement: S2 Fig — UV/Vis spectra of sarcosine (secondary amine) and glutamic acid (primary amine) with ninhydrin after heating at A) 100°C for 2 min, and B) 80°C for 2 min. (TIF) [file pone.0196010.s002.tif]

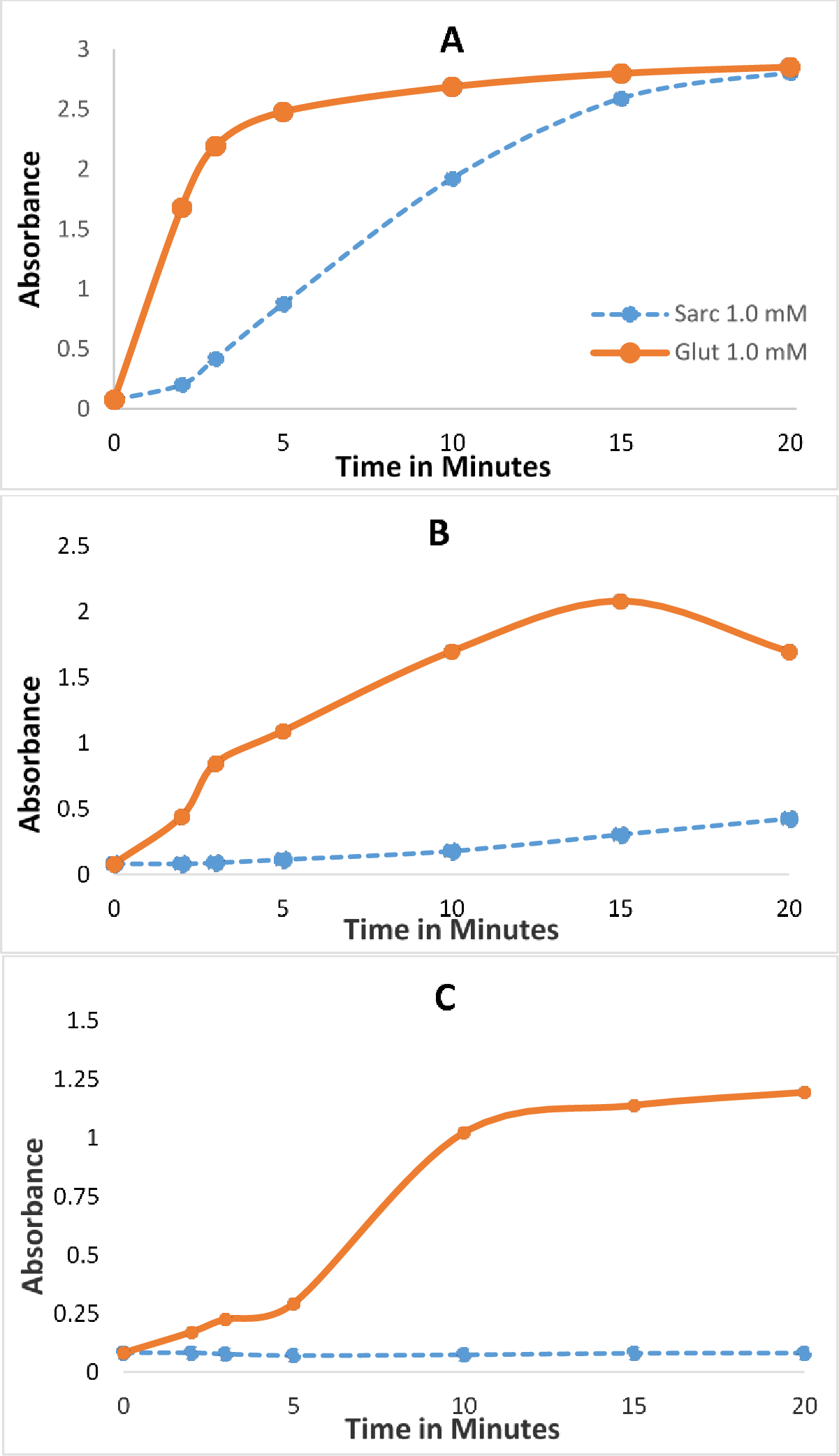

Supplement: S3 Fig — Time course plots of the development of primary amine or secondary amine with ninhydrin (A) at 100°C (B) vs 80°C and (C) vs 60°C. Glutamic acid was used as model primary amine, and sarcosine was used as a model secondary amine. (TIF) [file pone.0196010.s003.tif]

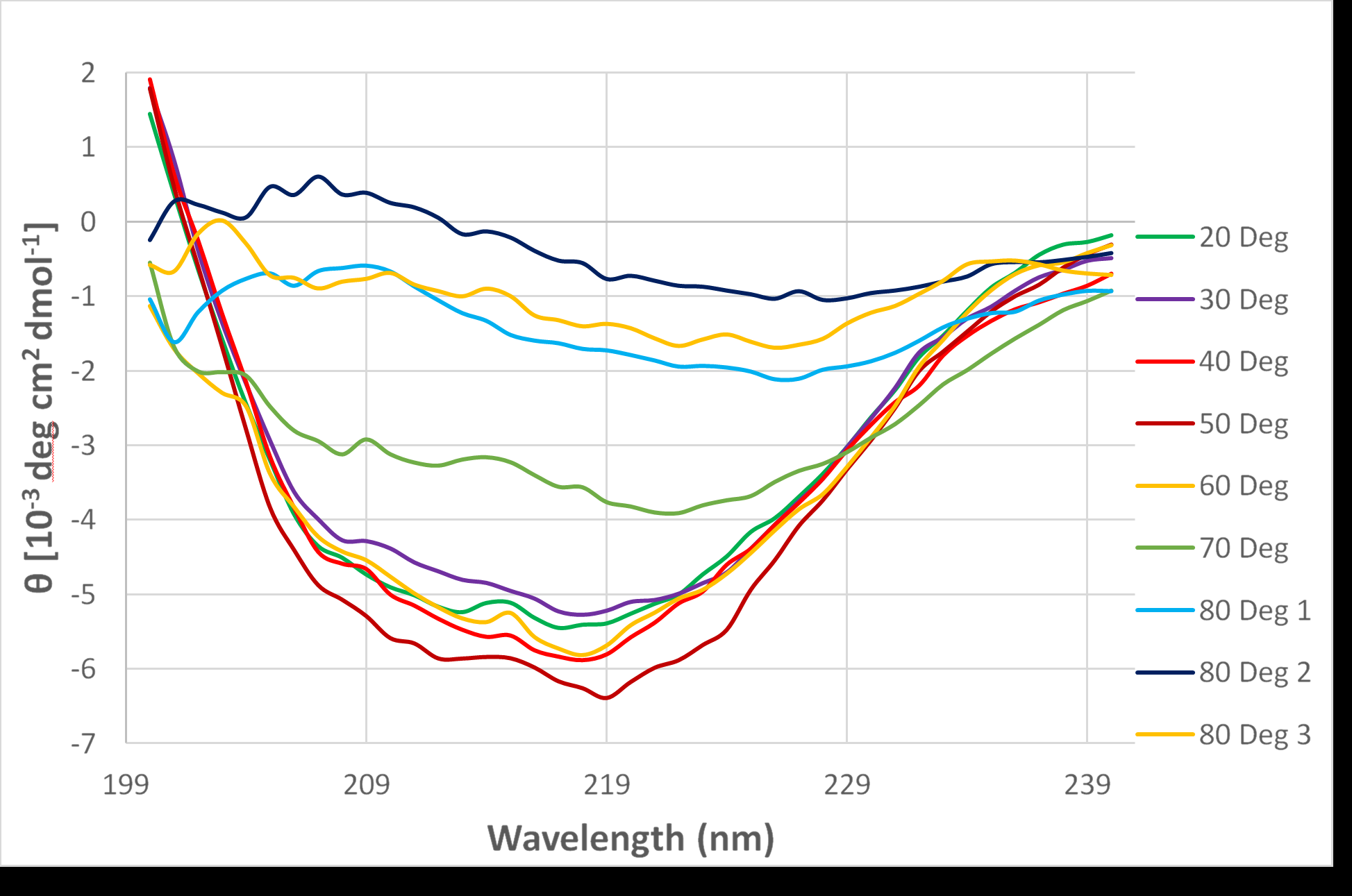

Supplement: S4 Fig — Circular Dichroism UV/Vis spectra of thermal denaturation of HiDapE observing the α-helical secondary structure. (TIF) [file pone.0196010.s004.tif]

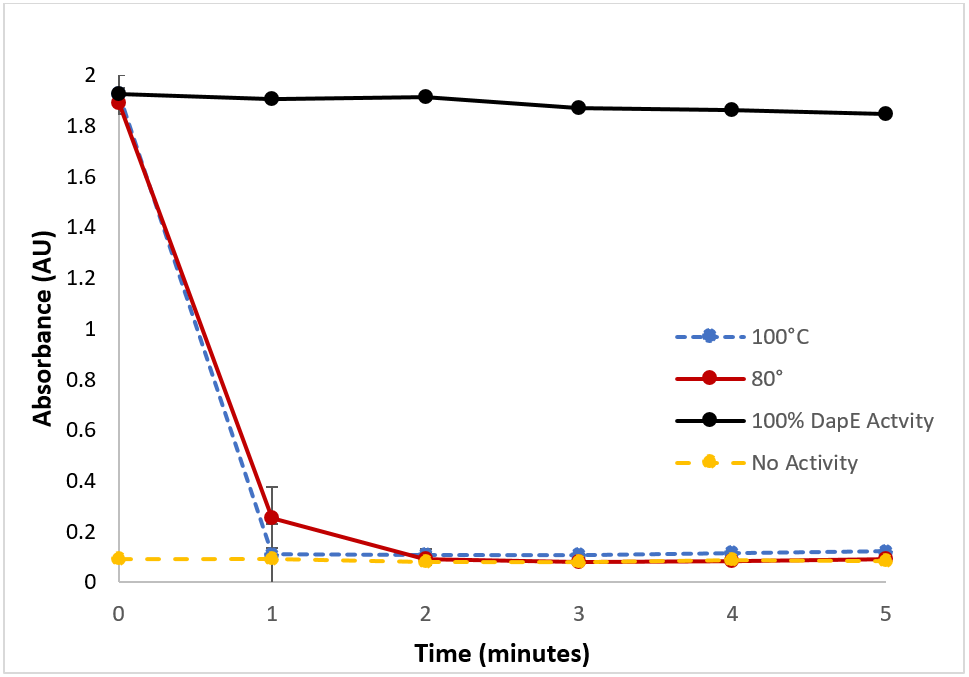

Supplement: S5 Fig — Pre-heat incubation reaction of HiDapE at 80°C vs 100°C compared to 100% enzymatic activity and 0% enzymatic activity. (TIF) [file pone.0196010.s005.tif]

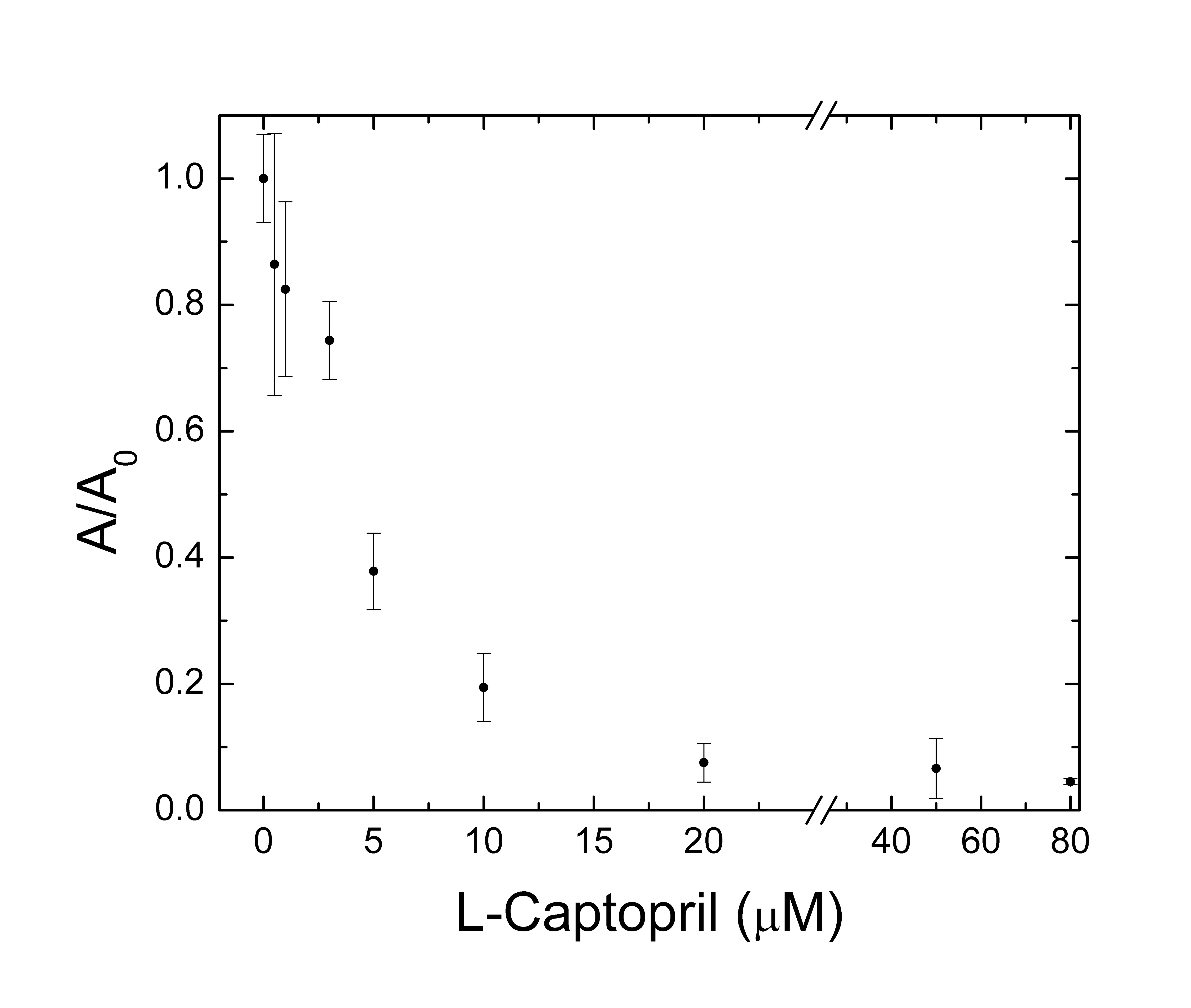

Supplement: S6 Fig — (TIF) [file pone.0196010.s006.tif]

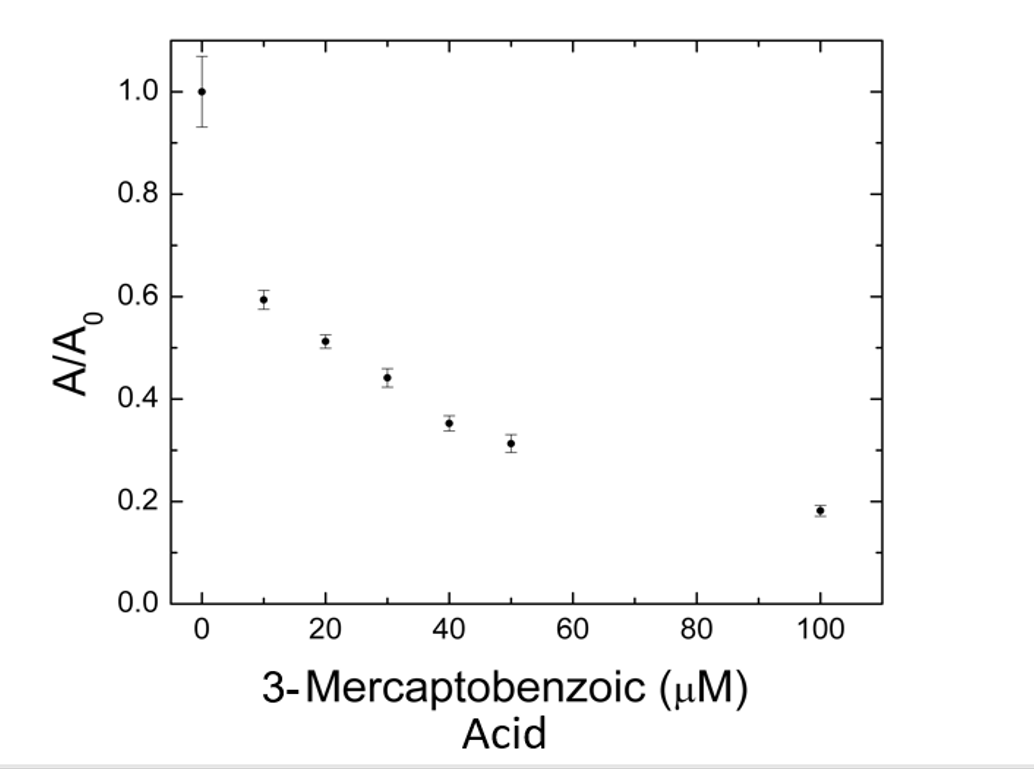

Supplement: S7 Fig — (TIF) [file pone.0196010.s007.tif]

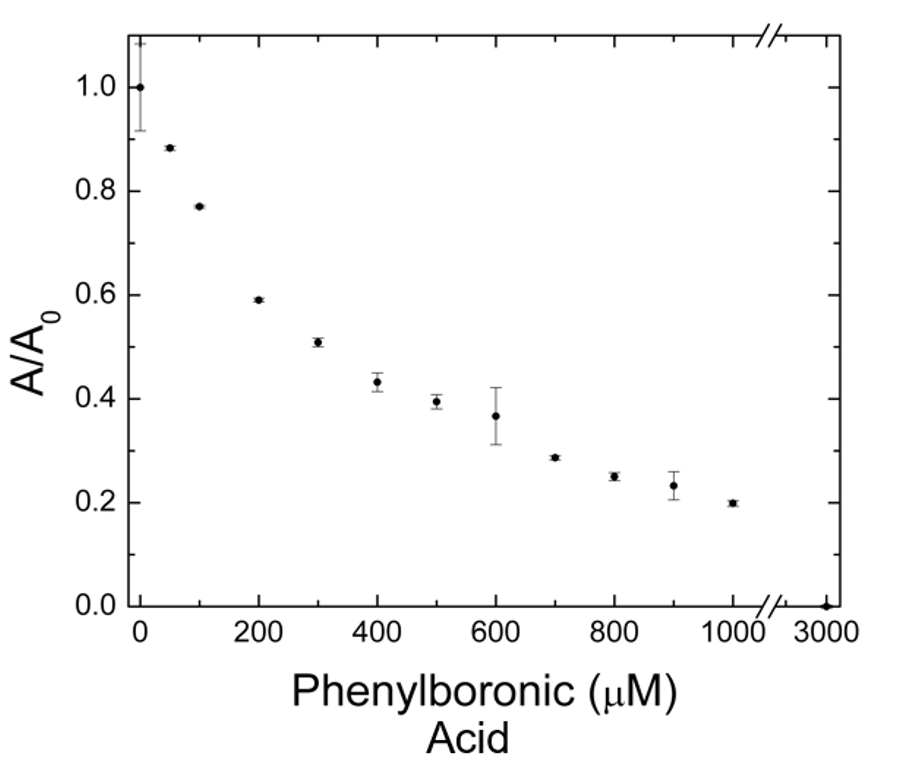

Supplement: S8 Fig — (TIF) [file pone.0196010.s008.tif]

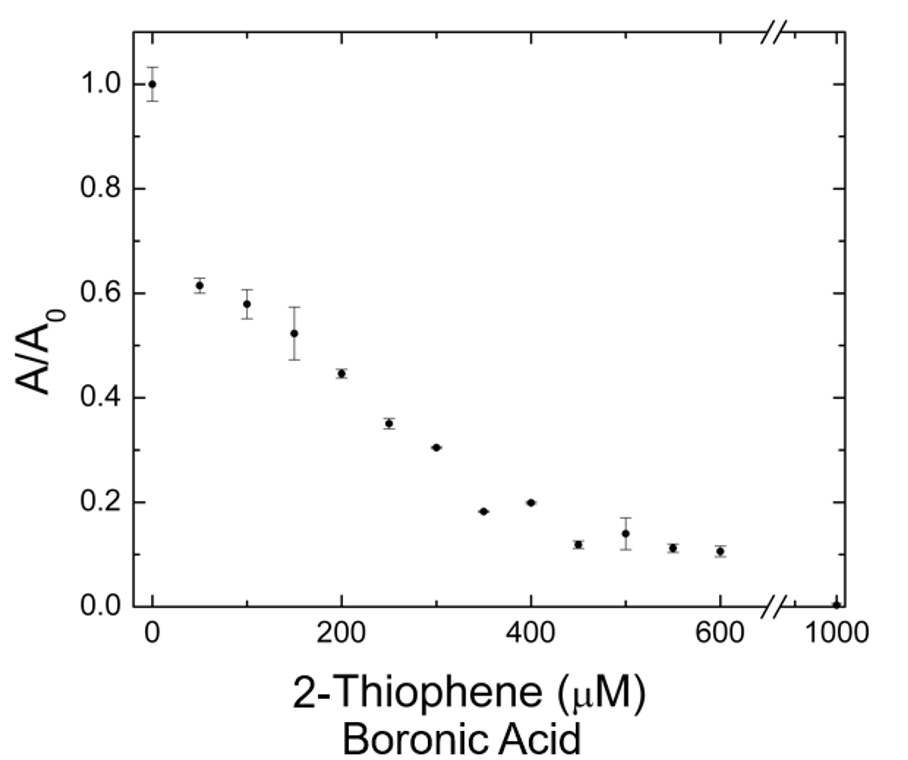

Supplement: S9 Fig — (TIF) [file pone.0196010.s009.tif]
